# TNKS1BP1 mediates AECII senescence and radiation induced lung injury through suppressing EEF2 degradation

**DOI:** 10.1186/s12931-024-02914-y

**Published:** 2024-08-07

**Authors:** Jiaojiao Zhu, Xingkun Ao, Yuhao Liu, Shenghui Zhou, Yifan Hou, Ziyan Yan, Lin Zhou, Huixi Chen, Ping Wang, Xinxin Liang, Dafei Xie, Shanshan Gao, Ping-Kun Zhou, Yongqing Gu

**Affiliations:** 1 Beijing Key Laboratory for Radiobiology, Beijing Institute of Radiation Medicine, Beijing, 100850 China; 2 Hengyang Medical College, University of South China, Hengyang, 421001 China; 3 College of Life Sciences, Hebei University, Baoding, 071001 China

**Keywords:** TNKS1BP1, Type II alveolar epithelial cells, Senescence, Radiation induced lung injury

## Abstract

**Background:**

Although recent studies provide mechanistic understanding to the pathogenesis of radiation induced lung injury (RILI), rare therapeutics show definitive promise for treating this disease. Type II alveolar epithelial cells (AECII) injury in various manner results in an inflammation response to initiate RILI.

**Results:**

Here, we reported that radiation (IR) up-regulated the TNKS1BP1, causing progressive accumulation of the cellular senescence by up-regulating EEF2 in AECII and lung tissue of RILI mice. Senescent AECII induced Senescence-Associated Secretory Phenotype (SASP), consequently activating fibroblasts and macrophages to promote RILI development. In response to IR, elevated TNKS1BP1 interacted with and decreased CNOT4 to suppress EEF2 degradation. Ectopic expression of EEF2 accelerated AECII senescence. Using a model system of TNKS1BP1 knockout (KO) mice, we demonstrated that TNKS1BP1 KO prevents IR-induced lung tissue senescence and RILI.

**Conclusions:**

Notably, this study suggested that a regulatory mechanism of the TNKS1BP1/CNOT4/EEF2 axis in AECII senescence may be a potential strategy for RILI.

**Supplementary Information:**

The online version contains supplementary material available at 10.1186/s12931-024-02914-y.

## Background

Radiation therapy is a crucial treatment approach for patients with lung cancer, breast cancer, esophageal cancer, and other thoracic tumors. The lung belongs to the moderate radiation-sensitive organ, and the radiation treatment of chest tumors can expose the lung tissue to a radiation dose beyond the threshold of its biological effect and lead to different degrees of radiation-induced lung injury (RILI) [1–4]. The high incidence of RILI limits the total dose of radiotherapy for tumors, which not only reduces the efficacy of radiotherapy but also significantly increases the risk of death in some severe RILI.

Because the specific pathogenesis of RILI is not very clear, there is a lack of effective disease prediction diagnosis and treatment indicators. At present, the clinical treatment of radiation pneumonia mainly uses hormone drugs, which have side effects such as reducing human immunity. In addition, because radiation induced pulmonary fibrosis (RIPF) is an irreversible process and a lack of efficient and low-toxicity treatment, the quality of patient life is seriously affected. Therefore, it is of great clinical value to study the progression from early blockade of RILI to RIPF, elucidate the underlying mechanisms of RILI development, and identify targets with potential therapeutic effects, which is of important clinical significance for the prevention and treatment of RILI.

Alveolar epithelial cells (AEC) are the key driving target cells of RILI, and their dysfunction is a key effector process in RILI. The alveolar epithelial cells are divided into type I alveolar epithelial cells (AECI), which constitute the main skeleton of the alveolar, and type II alveolar epithelial cells with proliferation and differentiation ability (AECII) [5–7]. AECII, can, on the one hand, supplement type I alveolar epithelial cells to maintain the alveolar structure, and on the other hand, undergo epithelial-stromal transformation (Epithelial-mesenchymal transition, EMT) to produce fibroblasts and participate in the impaired repair [8–10]. Therefore, AECII is important for maintaining the stability of alveolar tissue structure and function.

Studies have suggested undergo early apoptosis and sustained cellular senescence in AECII after IR [11, 12]. Senescent cells cause impaired tissue repair and Senescence-Associated Secretory Phenotype (SASP) that undergo characteristic changes including transcriptional, epigenetic, morphological, and metabolic alterations [13]. Once the cells undergo senescence, will highly express and release pro-inflammatory cytokines (interleukin-6 (IL-6); interleukin-1 β (IL-1β), etc.), growth factor (transforming growth factor-β (TGF-β); granulocyte–macrophage colony stimulating factor (GM-CSF) and chemokines (CXCL1/3/10) [12, 14, 15]. SASP-related pro-inflammatory cytokines have the biological effects of activating fibroblasts and macrophages [16, 17]. The activated fibroblasts can promote excessive deposition of ECM and aggravate pulmonary fibrosis; Meanwhile, SASP can protect myofibroblasts from apoptosis and can spread the senescence phenotype to the surrounding cells, and cause paracrine senescence [18–21]. The activation of macrophages causes the abnormal activation of the immune system to further produce a large number of pro-inflammatory cytokines, exacerbating lung inflammation and leading to pulmonary fibrosis. Therefore, the accumulation of senescent cells and secretion of SASP may become a new target for the treatment of tissue senescence lesions [22–26]. In conclusion, a lot of studies have been carried out on IR-induced RILI, but there are still few studies on the regulatory mechanism of senescence of AECII caused by IR.

TNKS1BP1, also known as TAB182, is a new radiation-responsive protein in our previous study. It is an anchor polymerase 1 (Tankyrase1) binding protein with a molecular weight of 182 KD and is widely found in the cell membrane, cytoplasm, and nucleus. TNKS1BP1 was found in a yeast two-hybrid experiment interacting between the human telomere repeat binding protein-1 (TRF1) and Tankyrase1 [27]. Previous studies suggested that tankyrase1 has 5 ARC (Ankyrin Repeat Cluster, ARC), recognition sites, including 3 ARC that can bind to TNKS1BP1, suggesting that TNKS1BP1 and TRF1, may compete for the binding of Tankyrase 1 and that Tankyrase1 is a positive regulator of telomere length [27]. Telomere length is closely related to cellular senescence and telomere shortening leads to cellular senescence [21, 28]. This suggests that TNKS1BP1 may have a similar biological function to TRF1, involved in the regulation of cellular senescence. In our early result about mass spectrometry analysis, eukaryotic translation elongation factor-2 (EEF2), as an important regulator of protein translation, interacted with TNKS1BP1. EEF2 is a GTP-dependent translocase that promotes ribosome translocation of peptidyl-tRNA during polypeptide elongation, widely involved in a variety of peptide chain elongation processes. It has been shown that EEF2 expression is significantly up-regulated in replication senescent cells [29–32]. Then TNKS1BP1 in the radiation-induced senescence in lung epithelial cells and whether it participates in AECII senescence by regulating EEF2 is not clear.

In this work, TNKS1BP1 was found to be highly expressed in IR-induced AECII and lung tissue of RILI mice. Importantly, the deletion of TNKS1BP1 significantly protected mice from RILI along with a marked reduction of senescence in the lung tissue. Mechanistically, TNKS1BP1 up-regulated EEF2 by maintaining its stability to induce the senescence of AECII, and CNOT4 might be a potential E3 ubiquitin ligase of EEF2 in this regulatory pathway. Therefore, targeting TNKS1BP1 will provide a novel theoretical and experimental basis for clinical prevention and treatment of RILI.

## Materials and methods

### Cell culture

The cell lines used in this study, A549, RAW264.7, and HLF-1, were cultured with DMEM medium, 10% FBS and 1% penicillin/streptomycin. In parallel, we used lentivirus to construct the A549 cell line with stable knockdown of TNKS1BP1. For radiation, A549 were cultured for 48 h after 10 Gy γ ray. For inhibitor treatment, A549 were transfected by si-EEF2 and si-CNOT4 with 100 nmol / L by lipo-2000 in a 60 mm culture dish. For over-expression, we used plasmid-TNKS1BP1 transfected A549 by PEI with 4 ug in a 60 mm culture dish. The fibroblasts and macrophages were co-cultured with the conditioned medium from the treated A549 cells.

### Mice and RILI model construction

Wild-type mice and TNKS1BP1^−/−^ mice (TNKS1BP1 knock out) are the C57BL/6 backgrounds, and were reproduced in our laboratory. All mice were bred and maintained at accredited animal facilities under specific-pathogen-free conditions in individually ventilated cages on a strict 12-h day-night cycle with a regular chow diet. The mice used in this study were 6–8 weeks old. Littermate mice were randomly grouped into control and treatment groups for all experiments in this study. All animal experiments were performed in compliance with the Guide for the Care and Use of Laboratory Animals, performed according to guidelines for the Laboratory Animal Guideline of Welfare and Ethics of China, and All experimental procedures were approved by the Animal Care and Use Committee at the Military Academy of Medical Sciences (IACUC-DWZX-2022–572).

To construct RILI model by chest irradiation with ^60^Co γ-rays of 20 Gy. Mice were divided into three groups: WT + NC (WT), WT + IR, and TNKS1BP1^−/−^ + IR (KO + IR) and lung tissues were collected at 1- and 2-months following exposing to IR.

### Histological analyze

The largest left lung lobe was excised, fixed in 4% paraformaldehyde, and cut into 5 μm thickness. Histopathological study was made using hematoxylin and eosin (H&E; Cat#ZLI-9610, ZSGB-BIO, Beijing, China) staining and quantitated by a semi-quantitative scoring system in Szapiel. Masson’s triple stain according to the Masson’s Trichrome Stain Kit manuscript (Cat#G1340, Solarbio Life Science, Beijing, China) was used to analyze the collagen. P21 protein expression in lung tissue was assessed by immunohistochemistry using the ZSGB-BIO Mouse two-step method kit. Images were acquired using Nikon’s Eclipse E600 microscope (Nikon, Tokyo, Japan) and all the figures were quantified using Image J software (Bethesda, MD, USA).

### qRT-PCR

RNA was isolated with Trizol reagent (Invitrogen). cDNA was synthesized using HIScript III RT super Mix for qPCR (VAZYME). qRT-PCR was performed by using Taq pro universal sybr qpcr master Mix (VAZYME), and reactions were run with QuantStudio Real-Time PCR software V1.3 (Thermo Fisher Scientific). The results are displayed as relative expression values normalized to β-actin. Primers used for qRT-PCR are listed in Supplementary Table 1.

### Immunoblot analysis

Cell was lysed in NETN100 and Tissue was lysed in protein extraction reagent (Thermo Fisher Scientific) containing protease inhibitor cocktail (Roche). Protein concentration was determined by BCA protein assay ((Beyotime). 30 μg of protein lysate per lane was run through 10% Tris–Glycine Gels and transferred to NC Membrane (KainuoBio, Beijing). The membrane was blocked for 1 h in 5% fat-free dried milk in Tris-buffered saline containing 0.1% Tween 20 (TBST) and incubated overnight with primary antibody at 4 °C. The membrane was then washed 3 times in TBST and incubated with an HRP-conjugated secondary antibody for 1 h at room temperature. Finally, the membranes were developed using the ECL Prime Western blot detection reagent. Antibodies used in the study are listed in Supplementary Table 2.

### Co-immunoprecipitation

Cells were lysed with lysis buffer (300 mM NaCl, 50 mM Tris–HCl (pH 8.0), 0.5% (v/v) NP-40, 1 mM EDTA, protease inhibitor cocktail (Roche)) at 4 °C about 30 min, centrifuged at 13,000 g for 10 min at 4 °C. Supernatants were collected and incubated with 1 μg primary antibody for 2 h at 4 °C. Protein A/G agarose (Santa Cruz, CA, US) was added for 4 h incubation at 4 °C. Washed agarose beads three times with lysate buffer. Immunoprecipitation complexes were collected and analyzed by Western blot assay.

### Protein profile

The co-immunoprecipitation was conducted to capture the protein interacted with TNKS1BP1. The LC–MS/MS (liquid chromatography-tandem mass spectrometry) was used to recognize and identify the interaction protein.

### Immunofluorescence staining

After deparaffinization, hydration, and washing, high-pressure antigen retrieval using citrate buffer. The 0.3% Triton X-100 in PBS used to permeate sections. Goat serum was used to block the sections and the primary antibodies were incubated overnight at 4 °C. Secondary antibodies were incubated for 2 h at 25 °C. Sections were mounted with the medium containing DAPI for fluorescence (ZSGB-BIO, Beijing, China). Images were scanned with a confocal immunofluorescence microscope (Carl Zeiss, Germany).

### SA-β-gal staining

SA-β-gal staining was conducted by the SA-β-gal staining kit (G1580, Solarbio). Briefly, cells were washed 3 times with PBS, and fixed with indicating fixation solution for 15 min at room temperature. Washing 3 times with PBS, the cells were incubated with 1 mL SA-β-gal staining solution overnight at 37 °C (CO_2_-free atmosphere). Images were scanned with microscope (Nikon, Japan).

### Statistical analysis

All experiments were performed with at least three replicates in each assay or with three independent experiments. Data were statistically analyzed by using IBM SPSS.20.0. Tests employed are mentioned in the figure legends. Data is shown as mean with standard error. *p* < 0.05 was considered statistically significant.

## Results

### Ionizing radiation can induce high expression of TNKS1BP1 and senescence of lung epithelial cells

**Fig. 1:**
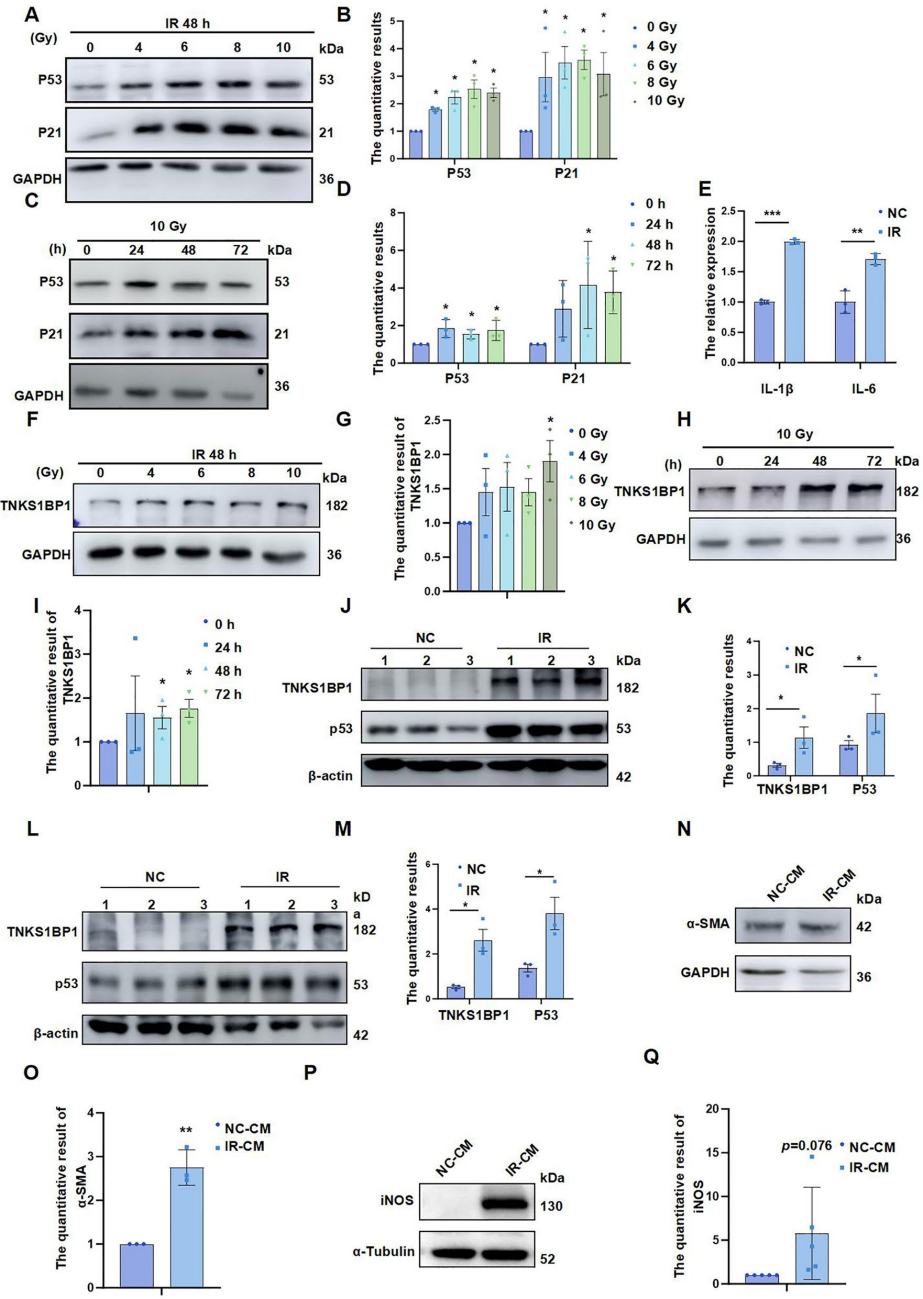
Ionizing radiation can induce high expression of TNKS1BP1 and senescence of AECII. (**A**), P21 and P53 expression level on AECII Cells with 0, 4, 6, 8, 10 Gy after 48 h. (**B**), the quantification of (A). (**C**), P21 and P53 expression level on AECII Cells at the indicated time points following exposing to 10 Gy IR. (**D**), the quantification of (C). (**E**), senescence-associated secretory phenotype (SASP) related inflammatory factors (IL-1β and IL-6) level on AECII Cells at 48 h following exposing to 10 Gy IR. (**F**), TNKS1BP1 expression level on AECII Cells with 0, 4, 6, 8, 10 Gy after 48 h. (**G**), the quantification of (F). (**H**), TNKS1BP1 expression level on AECII Cells at the indicated time points following exposing to 10 Gy IR. (**I**), the quantification of (H). (**J-M**), TNKS1BP1 and P53 expression level on lung tissue of 20 Gy single total thoracic local radiation—induced lung injury mice, (**J**), after radiation 1 month, (**K**), the quantification of (J), (**L**), after radiation 2 months, (**M**), the quantification of (L). (**N**), α-SMA expression level on HLF-1 Cells after co-cultured with conditioned medium from IR-induced AECII. (**O**), the quantification of (N). (**P**), iNOS expression level on RAW264.7 Cells after co-cultured with conditioned medium from IR-induced AECII. (**Q**), the quantification of (P). Data are presented as mean ± SD. For statistical analysis, unpaired Student’s *t* test, ^*^p < 0.05, ^**^p < 0.01, ^***^p < 0.001 mean the difference statistical significance

We treated lung epithelial cells with different doses of IR at different times and found that IR induced up-regulation of senescence marker proteins P21 and P53 expression in AECII (Fig. 1A-D). The SA-β-gal staining was used to detected the senescence of epithelial cells after 6 h, 12 h, 24 h, 48 h and 72 h following exposing to 10 Gy IR. Results showed that the SA-β-gal-positive cells significantly increased after 48 h and 72 h compared to 0 h (supplementary Fig. 1A and B), and increased expression of senescence-associated secretory phenotype IL-1β and IL-6 and these phenotypes were most apparent at 48 h after IR at 10 Gy (Fig. 1E and supplementary Fig. 1C). To further explore the molecular mechanism of IR-induced senescence in AECII, studies suggest that the binding sites of tankyrase1 and TNKS1BP1 is similar to the negative regulator TRF1, and that tankyrase1 is a positive regulator of telomere length. We found that TNKS1BP1 protein showed a time-dose-dependent increase after IR, most pronounced at 10 Gy 48 h (Fig. 1F-I). Moreover, in a mouse model of RILI constructed by 20 Gy γ rays, TNKS1BP1 and P53 expression increased in mouse lung tissue after IR (Fig. 1J-M), which further suggests the role of TNKS1BP1 in RILI.

RILI is the result of multicellular interaction, to clarify the effect of AECII senescence on fibroblasts and macrophages, we used IR-induced senescent AECII to co-culture with fibroblasts and macrophages separately and found that AECII senescence can promote fibroblast activation marker α-SMA (Fig. 1N and O) and macrophage activation marker iNOS expression (Fig. 1P and Q). The above results suggested that senescence of AECII can promote fibroblast and macrophage activation.

### IR promotes senescence of AECII induced by high expression of TNKS1BP1 could activate fibroblasts and macrophages

**Fig. 2:**
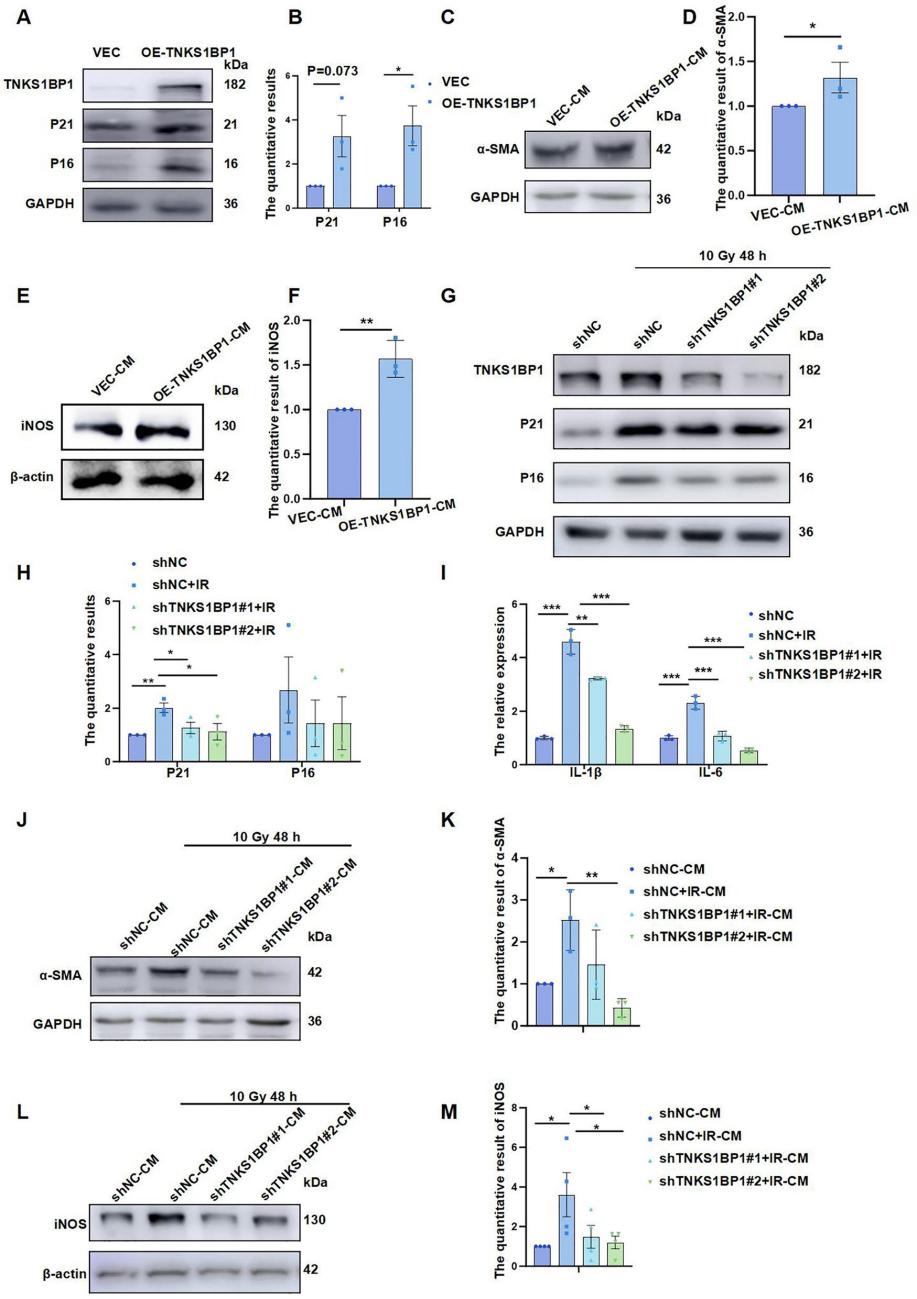
IR promotes senescence of AECII induced by high expression of TNKS1BP1 could activate fibroblasts and macrophages. (**A**), P21 and P16 expression level on AECII Cells after over-expression TNKS1BP1 with 4 μg plasmid transfected. (**B**), the quantification of (A). (**C**), α-SMA expression level on HLF-1 Cells after co-cultured with conditioned medium from overexpression TNKS1BP1 AECII. (**D**), the quantification of (C). (**E**), iNOS expression level on RAW264.7 Cells after co-cultured with conditioned medium from overexpression TNKS1BP1 AECII. (**F**), the quantification of (E). (**G**), P21 and P16 expression level on AECII Cells after inhibition of TNKS1BP1 simultaneously under IR. (**H**), the quantification of (G). (**I**), senescence-associated secretory phenotype (SASP) related inflammatory factors (IL-1β and IL-6) on ACEII Cells after inhibition of TNKS1BP1 simultaneously under IR. (**J**), α-SMA expression level on HLF-1 Cells after co-cultured with conditioned medium from AECII in different treatment. (**K**), the quantification of (J). (**L**), iNOS expression level on RAW264.7 Cells after co-cultured with conditioned medium from AECII in different treatment. (**M**), the quantification of (L). Data are presented as mean ± SD. For statistical analysis, B, D and F were conducted by unpaired Student’s *t* test; H, I, K and M were conducted by One-way ANOVA, ^*^p < 0.05, ^**^p < 0.01, ^***^p < 0.001 mean the difference statistical significance

Then whether TNKS1BP1 can participate in the regulation of senescence in AECII, we found that the over-expression of TNKS1BP1 could promote P21 and P16 protein expression (Fig. 2A and B). IL-6 level increased in the supernatants of TNKS1BP1 overexpression A549 cells (supplementary Fig. 1D). Meanwhile, AECII over-expressing TNKS1BP1 were co-cultured with fibroblasts and macrophages separately, and we found that AECII senescence induced high expression of fibroblast activation marker α-SMA (Fig. 2C and D) and high expression of macrophage marker iNOS (Fig. 2E and F). These findings confirmed that TNKS1BP1 modulates AECII senescence, further to clarify whether TNKS1BP1 is involved in IR-induced AECII senescence. We found that stable knockdown of TNKS1BP1 inhibited the increase of P21 and P16 proteins induced by IR (Fig. 2G and H) and decreased the increase of SASP-related inflammatory factors IL-1β and IL-6 induced by IR (Fig. 2I). Additionally, knockdown of TNKS1BP1 also could decrease the percentage of SA-β-gal-positive cells (supplementary Fig. 1 E and F). Moreover, after stable knockdown of TNKS1BP1 under IR simultaneously inhibited the senescence phenotype of AECII, fibroblast (Fig. 2J and K) and macrophage (Fig. 2L and M) activation induced by AECII senescence were also significantly suppressed. These results indicated that targeting TNKS1BP1 for inhibition of AECII senescence may suppress fibroblast and macrophage activation during RILI, thus effectively alleviating RILI.

### Under IR, high TNKS1BP1 expression induces AECII senescence through up-regulation of EEF2

**Fig. 3:**
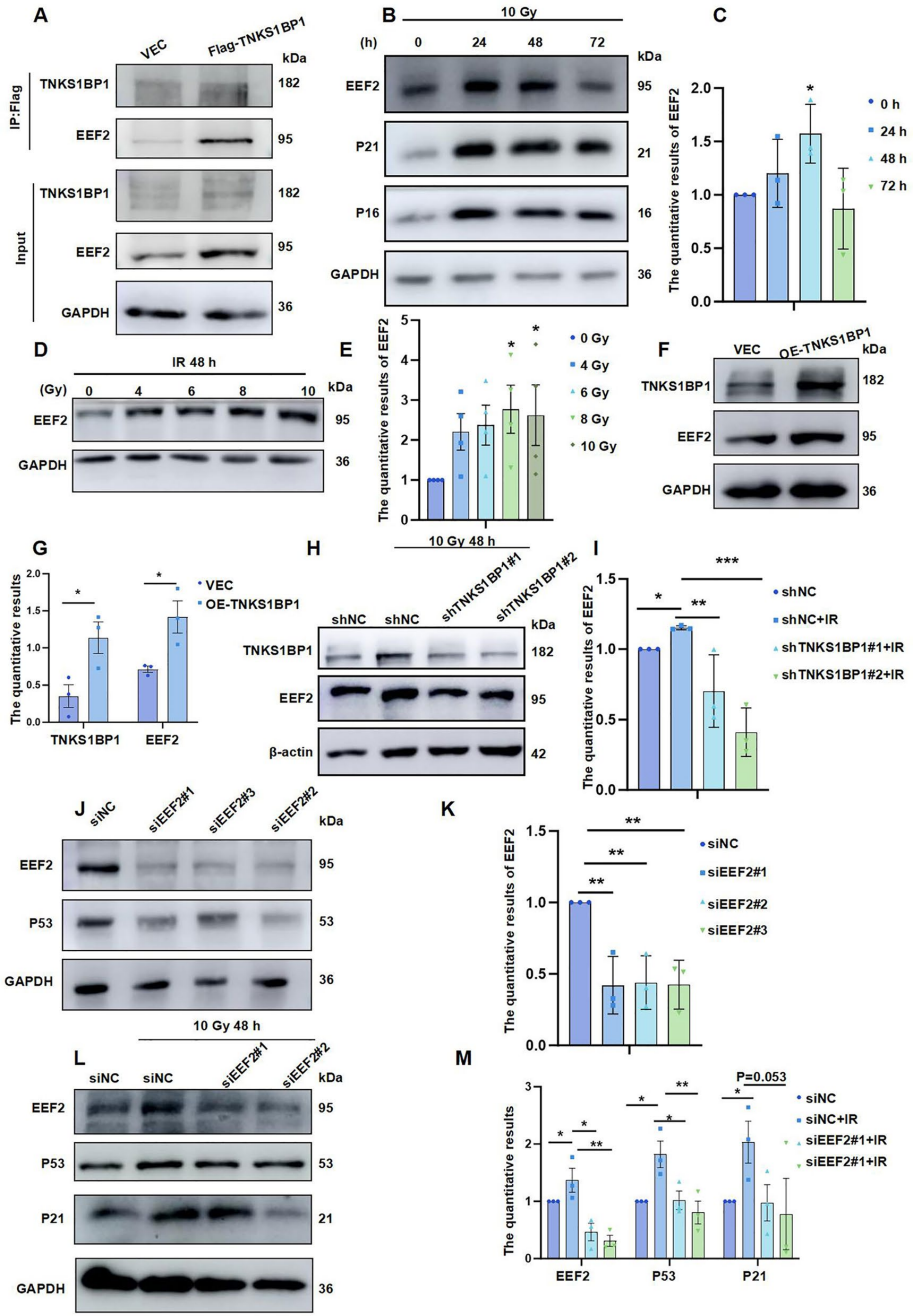
Under IR, high TNKS1BP1 expression promoted AECII senescence through up-regulation of EEF2. (**A**), the interaction of TNKS1BP1 and EEF2 on AECII Cells identified by co-IP. (**B**), EEF2 expression level on AECII Cells with 0, 4, 6, 8, 10 Gy after 48 h. (**C**), the quantification of (B). (**D**), EEF2 expression level on AECII Cells at the indicated time points following exposing to 10 Gy IR. (**E**), the quantification of (D). (**F**), EEF2 expression level on AECII Cells after over-expression TNKS1BP1 with 4 μg plasmid transfected. (**G**), the quantification of (F). (**H**), EEF2 expression level on ACEII Cells after inhibition of TNKS1BP1 simultaneously under IR. (**I**), the quantification of (H). (**J**), P53 expression level on ACEII Cells after inhibition of EEF2. (**K**), the quantification of (J). (**L**), P53 and P21 expression level on ACEII Cells after inhibition of EEF2 simultaneously under IR. (**M**), the quantification of (L). Data are presented as mean ± SD. For statistical analysis, C, E and G were conducted by unpaired Student’s *t* test; I, K and M were conducted by One-way ANOVA, ^*^p < 0.05, ^**^p < 0.01, ^***^p < 0.001 mean the difference statistical significance

To study the molecular mechanism by which TNKS1BP1 promotes the senescence of AECII, we used protein profiling to screen the interacting proteins of TNKS1BP1 and found that the eukaryotic translation elongation factor (EEF2), which can regulate the expression of the p53 gene, interacts with TNKS1BP1 (data not shown). Co-IP assay demonstrated the interaction of TNKS1BP1 with EEF2 (Fig. 3A). Notably, EEF2 increased in a time- and dose-dependent manner under IR (Fig. 3B-E). Moreover, over-expression of TNKS1BP1 promoted EEF2 protein expression (Fig. 3F and G). Under IR, Knockdown of TNKS1BP1 effectively suppressed the high expression of EEF2 caused by IR, suggesting that EEF2 may be involved in IR-induced AECII senescence (Fig. 3H and I). Further investigation revealed that inhibiting EEF2 significantly down-regulated the protein expression of the senescence-related protein P53 (Fig. 3J and K). Simultaneously, knockdown of EEF2 under IR resisted the increase of the senescence-associated protein P21 induced by IR (Fig. 3L and M). These above results confirmed that the elevated TNKS1BP1 expression could induce senescence of AECII by promoting EEF2 protein expression under IR.

### Low expression of CNOT4 promotes EEF2-mediated senescence of AECII in IR

**Fig. 4:**
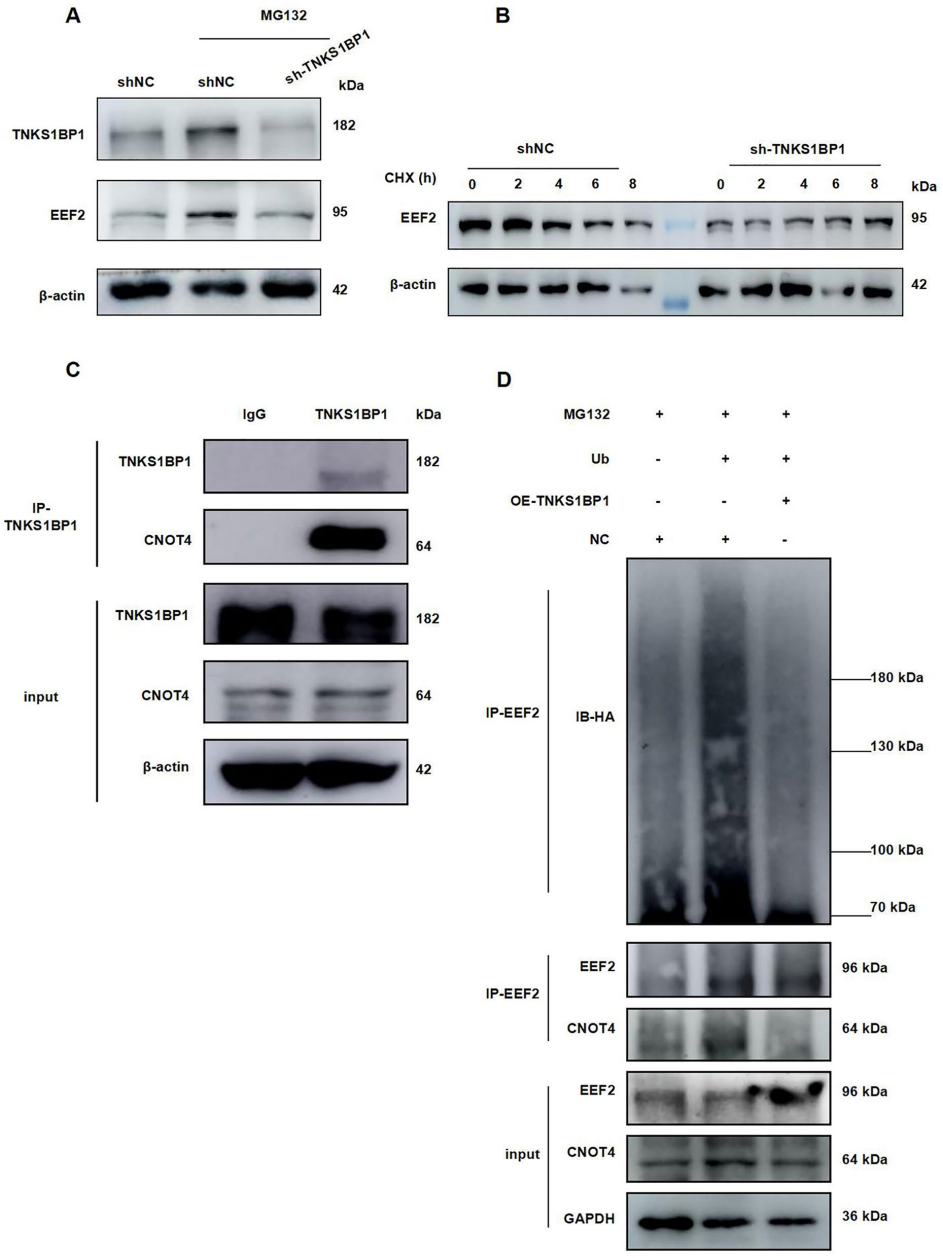
Over-expression TNKS1BP1 promoted EEF2 degradation. (**A**), EEF2 expression level on AECII Cells after inhibition TNKS1BP1 and treated with MG132. (**B**), EEF2 expression level on AECII Cells after inhibition TNKS1BP1 and treated with Cycloheximide (CHX). (**C**) co-IP assay analyzing the interaction of TNKS1BP1 and CNOT4. (**D**), co-IP assay analyzing the interaction of EEF2 and CNOT4, and the ubiquitin degradation of EEF2 after over-expression TNKS1BP1

The mechanism by which TNKS1BP1 promotes EEF2 protein expression remains unclear. To investigate whether TNKS1BP1 is involved in regulating EEF2 protein degradation in AECII cells, we performed experiments utilizing the proteasome inhibitor MG132 and CHX. Our results showed that TNKS1BP1 knockdown led to a reduction in EEF2 accumulation induced by MG132 (Fig. 4A), while TNKS1BP1 knockdown enhanced EEF2 degradation following CHX stimulation of AECII cells (Fig. 4B). Moreover, we found that CNOT4 could be a potential E3 ubiquitin ligase for EEF2, and the interaction of TNKS1BP1 and CNOT4 was confirmed by co-IP assay (Fig. 4C). Ubiquitination experiment was conducted to investigate how TNKS1BP1 regulates the degradation of EEF2 through ubiquitination. The results demonstrated that the overexpression of TNKS1BP1 inhibits the ubiquitin-mediated degradation of EEF2 (Fig. 4D).

**Fig. 5:**
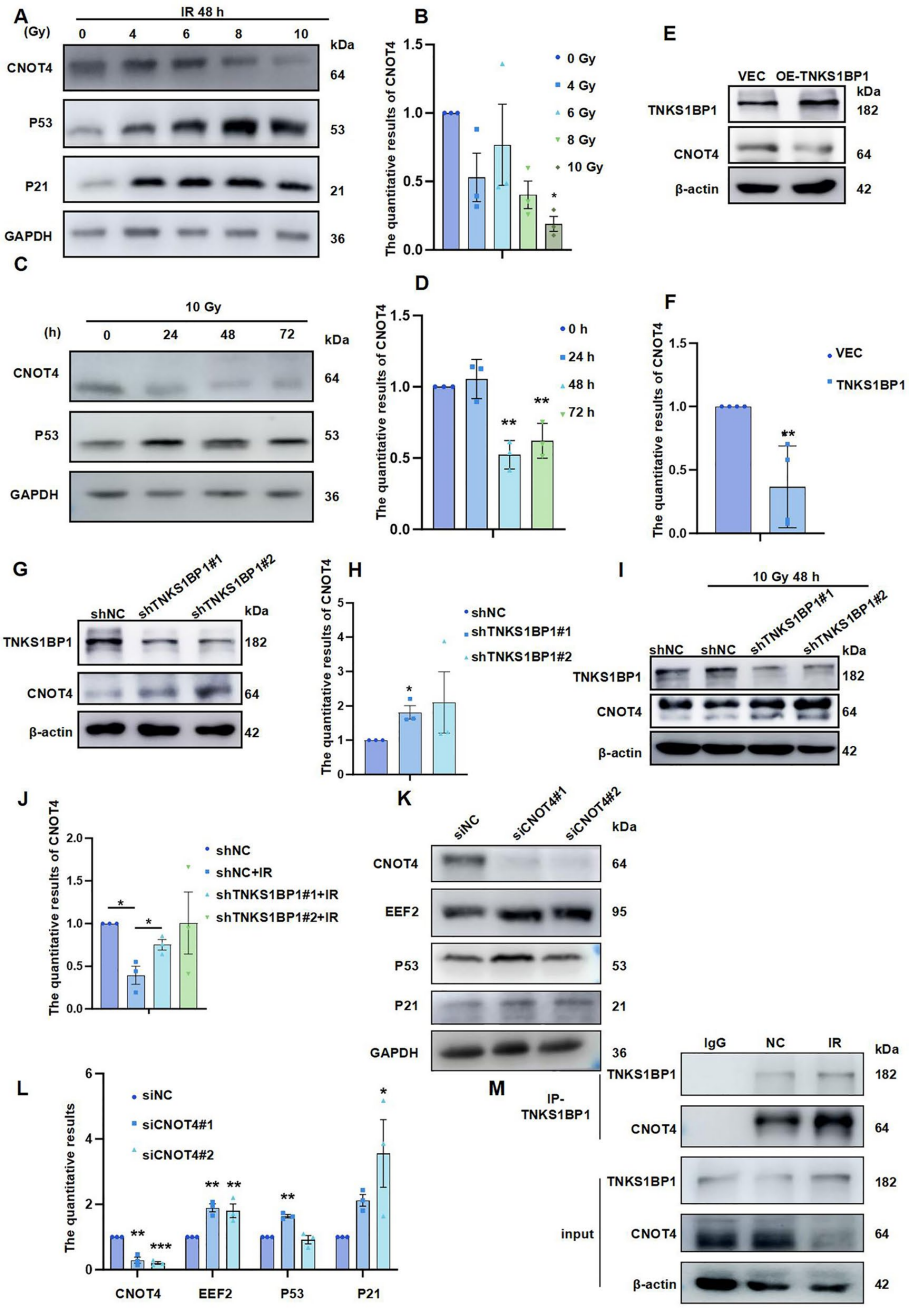
Low expression of CNOT4 induced EEF2-mediated senescence of AECII in IR. (**A**), CNOT4 expression level on AECII Cells with 0, 4, 6, 8, 10 Gy after 48 h. (**B**), the quantification of (A). (**C**), CNOT4 expression level on AECII Cells at the indicated time points following exposing to 10 Gy IR. (**D**), the quantification of (C). (**E**), CNOT4 expression level on AECII Cells after over-expression TNKS1BP1 with 4 μg plasmid transfected. (**F**), the quantification of (E). (**G**), CNOT4 expression level on ACEII Cells after inhibition of TNKS1BP1. (**H**), the quantification of (G). (**I**), CNOT4 expression level on ACEII Cells after inhibition of TNKS1BP1 simultaneously under IR. (**J**), the quantification of (I). (**K**), EEF2, P53, and P21 expression level on ACEII Cells after inhibition of CNOT4. (**L**), the quantification of (K). (**M**), co-IP assay analyzing the interaction of TNKS1BP1 and CNOT4 after radiation. Data are presented as mean ± SD. For statistical analysis, B, D, F, H and L were conducted by unpaired Student’s *t* test; J was conducted by One-way ANOVA, ^*^p < 0.05, ^**^p < 0.01, ^***^p < 0.001 mean the difference statistical significance

Meanwhile, we found that the protein expression of CNOT4 showed a time and dose-dependent reduction under ionizing radiation (Fig. 5A-D), which was most obvious at 48 h after 10 Gy. Furthermore, the protein content of CNOT4 decreased after the over-expression of TNKS1BP1 (Fig. 5E and F); while the CNOT4 protein content increased after the TNKS1BP1 knockdown (Fig. 5G and H). These results indicated that the content of CNOT4 is regulated by TNKS1BP1. Consequently, whether TNKS1BP1 can play a role in the regulation of CNOT4 content under IR.

We found simultaneous knockdown of TNKS1BP1 under IR that TNKS1BP1 knockdown resisted CNOT4 low expression caused by IR (Fig. 5I and J). Low expression of CNOT4 can promote EEF2 protein expression, and senescence related protein P53 and P21 also increased in AECII (Fig. 5K and L). Meanwhile, co-IP assay indicated the interaction between CNOT4 and TNKS1BP1 was increased after IR (Fig. 5M). The above findings suggested that CNOT4 may be involved in EEF2 expression regulation.

### Deficiency of TNKS1BP1 effectively alleviate RILI and lung tissue senescence

**Fig. 6:**
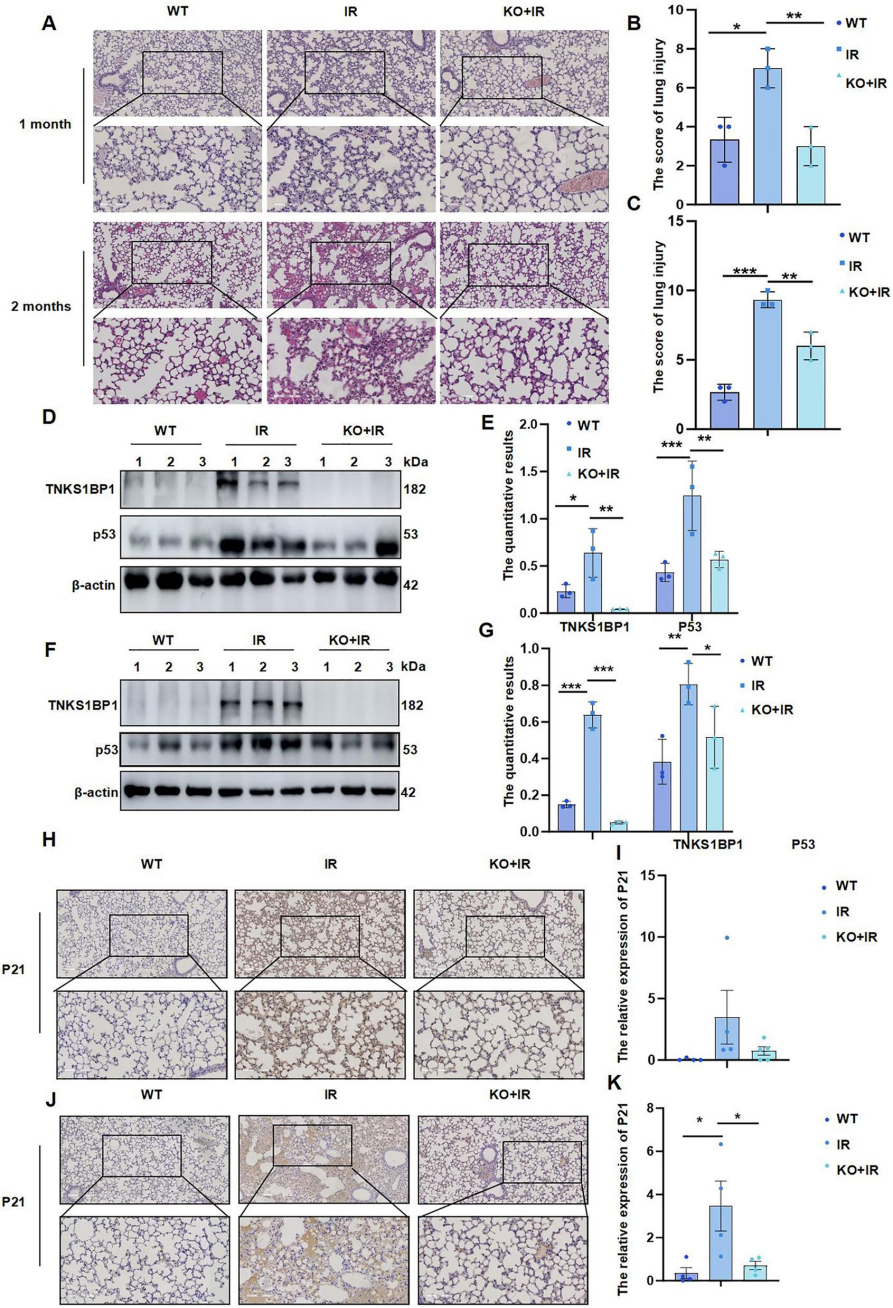
Deficiency of TNKS1BP1 effectively alleviated RILI and lung tissue senescence. (**A**) HE staining was performed to evaluate the RILI. Scale bars, 200 mm (up) and 100 mm (down). (**B**), quantitative of lung injury from HE at 1 month. (**C**), quantitative of lung injury from HE at 2 months. (**D**), TNKS1BP1 and P53 expression level on lung tissue of wild type mice and TNKS1BP1^−/−^ mice after 1 month following exposing to IR. (**E**), the quantification of (D). (**F**), TNKS1BP1 and P53 expression level on lung tissue of wild type mice and TNKS1BP1^−/−^ mice after 2 months following exposing to IR. (**G**), the quantification of (F). (**H-J**), immunohistochemistry was used to detect P21 expression level on lung tissue of wild type mice and TNKS1BP1^−/−^ mice at indicated time following exposing to IR, (**H**), after radiation 1 month. (**I**), quantitative of (H). (**J**), after radiation 2 months. (**K**), quantitative of (J). Data are presented as mean ± SD. For statistical analysis, One-way ANOVA, ^*^p < 0.05, ^**^p < 0.01, ^***^p < 0.001 mean the difference statistical significance

To clarify the feasibility of targeting TNKS1BP1 to therapy RILI, TNKS1BP1 gene knockout mice were generated. Subsequently, a RILI mouse model was established by exposing the mice to a single dose of 20 Gy γ-rays targeted to the total thoracic region, with assessments conducted at 1 month and 2 months post-irradiation. The results of increased inflammatory cell infiltration and alveolar collapse in lung tissue under IR showed that radiation induced acute pneumonia and injury (Fig. 6A-C). The knockout of TNKS1BP1 effectively alleviated the increased inflammatory cell infiltration and alveolar collapse induced by IR (Fig. 6A-C). Moreover, compared with WT mice, TNKS1BP1 knock out mice showed a decrease of collagen deposition in lung tissues after IR (supplementary Fig. 2 A-D). Whether TNKS1BP1 involved in the regulation of RILI by alleviating lung epithelial cell senescence? In response to IR, a significant increase in P53 protein levels was noted, which was markedly suppressed in mice lacking TNKS1BP1 (Fig. 6D-G), In lung tissue of RILI mice, there was a high expression of both P21 and P16 observed, and knockout of TNKS1BP1 substantially reduced this high expression caused by IR (supplementary Fig. 2E-F). Immunohistochemical staining showed that the increase of P21 induced by IR was reduced in TNKS1BP1 knockout mice (Fig. 6H-K). In the lung tissue of RILI mice, there was an observed increase in P21 levels alongside an increased co-localization of P21 and SFTPC, both of which were effectively inhibited by TNKS1BP1 knockout (supplementary Fig. 3A and B). Furthermore, the Immunofluorescence analysis was carried out on lung tissue to examine the senescence of epithelial cells and the activation of macrophages. It was also found that deficiency of TNKS1BP1 could effectively inhibit the expression of the iNOS (supplementary Fig. 2E and F) and decrease the co-localization of iNOS and F4/80 (supplementary Fig. 4A and B) induced by IR in lung tissue, suggesting that the deletion of TNKS1BP1 could effectively alleviate macrophages activation induced by lung tissue senescence. The above results indicated that TNKS1BP1 has the feasibility of targeted treatment of RILI.

## Discussion

The mechanism of RILI occurrence and development is highly intricate. Lung epithelial cells are the key initiating cells of RILI. Studies have confirmed that the apoptosis of lung epithelial cells in the early stage of injury, and the persistent presence of lung epithelial cell senescence can secrete senescence-related secretory phenotype (SASP). SASP can activate fibroblasts and macrophages, promoting RILI. Study have observed significant increases in markers of M1 macrophages from 1 to 5 months and in markers of M2 macrophages at 4 and 5 months on a RILI mice post-exposure to 17 Gy radiation. These findings suggest a crucial involvement of macrophage activation in the pathogenesis of RILI. What’s more, it is indicated that M1 and M2 macrophages may play varying roles in the progression of RILI [33]. Further study is needed to understand the mechanism by which lung epithelial cell senescence activates macrophages. In addition, studies have proven that RILI can be effectively alleviated by removing senescent cells [23, 25]. These studies suggested that lung epithelial cell senescence is an important reason for the occurrence and development of RILI, but its role and specific molecular mechanism in RILI are not clear. Therefore, it is of great importance to analyze how IR triggers the development of senescence in lung epithelial cells.

TNKS1BP1 was originally identified in its interaction with terminal anchor polymerase 1 (Tankyrase 1), but its specific biological role is not clear. Previous studies have suggested that TNKS1BP1 and TRF1 may compete for the binding of Tankyrase 1 [27]. In addition, TNKS1BP1 is distributed in both cytoplasm and nuclei, especially notably, TNKS1BP1 co-localizes with heterochromatin protein (HP1), suggesting the universality of its function. Until now, studies on TNKS1BP1 mainly focused on regulation tumor radioresistance and tumorigenesis [34–36]. In our previous study, TNKS1BP1 was identified as a new radiation-responsive protein, and its expression was increased after IR, suggesting that TNKS1BP1 may have an important role in the tissue damage induced by IR. In this study, TNKS1BP1 knockout mice was utilized to investigate its effects on relieving RILI and inhibiting the senescence of lung epithelial cells and lung tissues. In view of the molecular mechanism of TNKS1BP1 in regulating lung tissue senescence, we screened based on previous mass spectrometry results and combined cell and molecular experiments, we elucidated the molecular mechanism by which TNKS1BP1 regulates lung tissue senescence. Our findings identified that EEF2 plays an important role in the TNKS1BP1 high expression- induced lung epithelial cell senescence following exposure to IR.

Previously found that EEF2 regulates P53 protein expression and that P53 acts as a key senescence regulatory protein [37, 38], it was not clear whether EEF2 is involved in IR-induced senescence of lung epithelial cells. Our data confirmed for the first time that high expression of EEF2 under IR can promote senescence in lung epithelial cells. The molecular mechanism by which TNKS1BP1 is highly expressed and promotes the increase of EEF2 protein content under IR remains elusive. It has been confirmed that the protein content of EEF2 is regulated by ubiquitination. Consistent with the above-mentioned studies, our results indicated that TNKS1BP1 knockdown suppresses the accumulation of EEF2 induced by MG132 in lung epithelial, indicating that TNKS1BP1 may affect the protein stability of EEF2 involved in its content regulation.

However, it is not clear how TNKS1BP1 affects the protein stability of EEF2. We used bioinformatics means to screen the potential E3 ubiquitin ligase of EEF2, and found that CNOT4, as a potential E3 ubiquitin ligase of EEF2, may interact with TNKS1BP1. Previous studies have confirmed that the E3 ubiquitin ligase activity of CNOT4 can participate in the regulation of ubiquitination levels of multiple proteins [39]. To demonstrate the regulation of CNOT4 on EEF2, we confirmed the presence of CNOT4 interaction with both EEF2 and TNKS1BP1 by co-IP experiments, and knockdown of CNOT4 increased EEF2 protein content. These data indicate that CNOT4 can regulate the protein expression of EEF2. Additionally, CNOT4 showed a temporal dose-dependent reduction of under IR, and TNKS1BP1 knockdown under IR up-regulated CNOT4 low expression induced by IR and increased EEF2 protein content. Our results confirmed the regulation of EEF2 protein stability mediated due to changes in CNOT4 content under IR. And we firstly identified the radiation response of CNOT4. However, whether IR can affect the E3 ubiquitin ligase activity of CNOT4 needs to be further clarified, and the specific molecular mechanism of CNOT4 regulation by TNKS1BP1 also deserves further exploration.

**Fig. 7:**
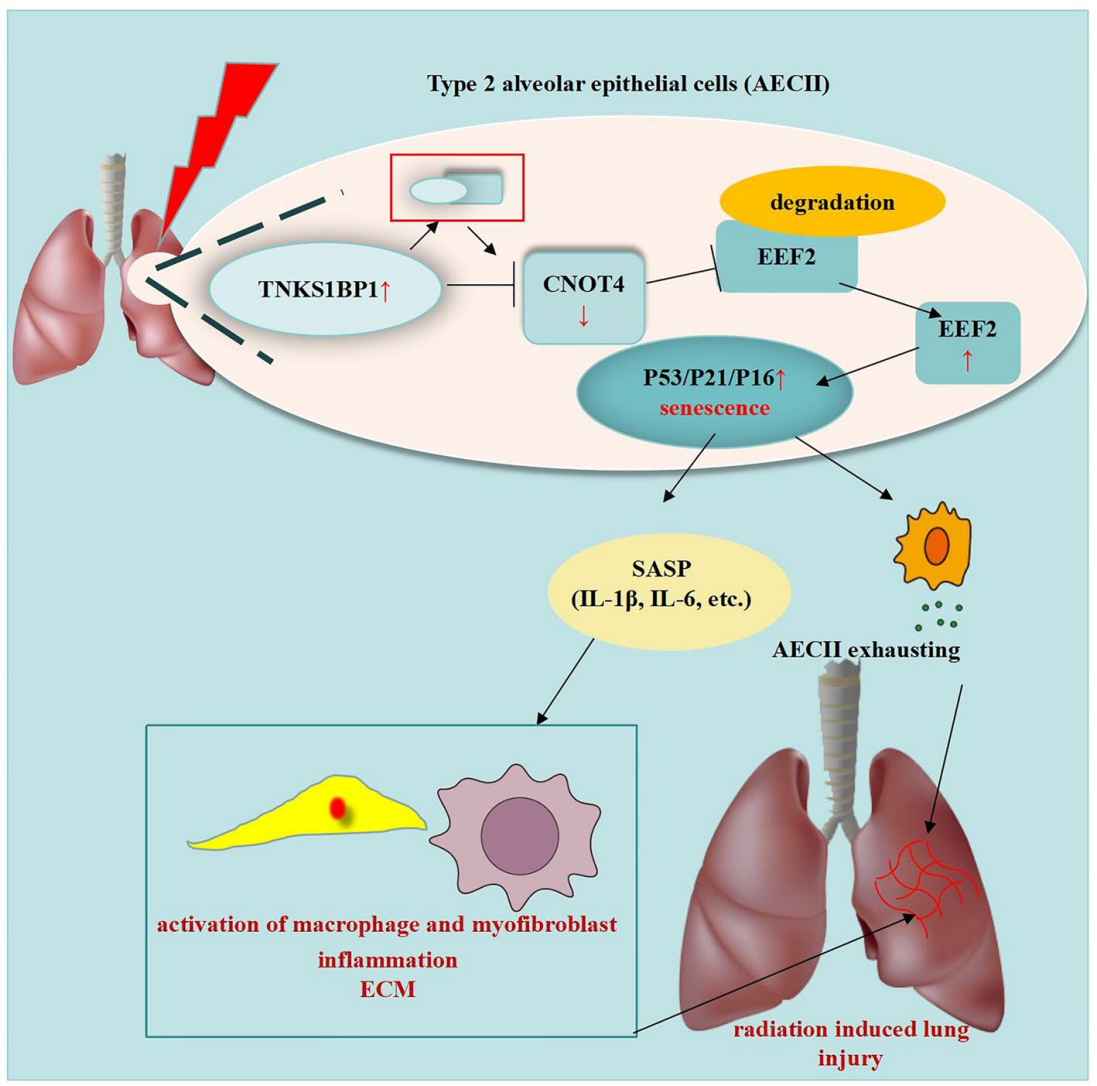
A proposed model of the role of TNKS1BP1/CNOT4/EEF2 pathway on AECII senescence and RILI. Under radiation, TNKS1BP1 directly interacted with CNOT4 and down-regulates its expression, thereby inhibit the degradation of EEF2, facilitates AECII senescence, finally induce the RILI

In summary, the present study provides novel insights into the key role of TNKS1BP1 in regulating the occurrence and progression of RILI. This is achieved through the CNOT4-mediated control of EEF2 expression and subsequent activation of the AECII senescence (Fig. 7). However, further in-depth analyses are critical for revealing the complete TNKS1BP1-associated regulatory network in RILI. First, targeting AECII senescence may provide valuable guidance for the development of novel preventive and therapeutic strategies, as current clinical therapeutic approaches are insufficient to prevent RILI. Given the number of specific inhibitors targeting AECII senescence is limited, and screening effective inhibitors against AECII senescence merits further investigation. D + Q (dasatinib + quercetin), a highly targeted drug that eliminates senescent cells, has been shown to be highly effective in ameliorating pulmonary fibrosis in numerous in *vitro* experiments and in mouse models of pulmonary fibrosis [23]. Therefore, starting from the targeted inhibition of TNKS1BP1, further studies investigating the inhibitor of senescence in RILI are highly warranted. Second, the mechanism of IR-induced senescence requires its selective recognition in many aspects. Notably, the DNA or telomere damage induced by radiation. In our study, TNKS1BP1 was positively correlated with senescence and other studies have investigated whether TNKS1BP1 can bind with TRF1, a negative regulator of telomere length; thus, we only focused that TNKS1BP1 regulated EEF2 to participate in AECII senescence, and whether TNKS1BP1 directly regulate telomere may be explored in the future. Third, further investigation is required to recognize the specific mechanism by which TNKS1BP1 regulates CNOT4. In addition, TNKS1BP1 may also participate in the other cell fate-associated regulatory network, which can’t be ruled out and needs further verification.

### Supplementary Information

Additional file 1.

Additional file 2: Supplementary Figure 1. The TNKS1BP1 promote IR induced AECII senescence. (A). SA-β-gal staining was performed on A549 cells at different time points (0 h, 6 h, 12 h, 24 h, 48 h, and 72 h) after exposure to 10 Gy of radiation; (B) The proportion of SA-β-gal-positive cells; (C). IL-6 levels detected by ELISA in the supernatants of A549 cells at 48 h after exposure to 10 Gy of radiation; (D) IL-6 levels detected by ELISA in the supernatants of A549 cells at 48 h after over-expression of TNKS1BP1. (E). SA-β-gal staining of A549 cells at each group; (F) The proportion of SA-β-gal-positive cells. Data are presented as mean ±SD. For statistical analysis, B, C and D were conducted by unpaired Student’s t test; F was conducted by One-way ANOVA; *p<0.05, ***p<0.001 mean the difference statistical significance.

Additional file 3: Supplementary Figure 2. TNKS1BP1 deletion ameliorates the IR induced lung tissue senescence. (A) Masson’s staining of lung tissues at 1 month after IR. Scale bar, 200 μm or 100 μm. (B) Collagen quantification by Masson’s trichrome staining of lung tissues with ImageJ software. (C) Masson’s staining of lung tissues at 2 months after IR. Scale bar, 200 μm or 100 μm. (D) Collagen quantification by Masson’s trichrome staining of lung tissues with ImageJ software. (E) iNOS, α-SMA, P21 and P16 expression level on lung tissue of wild type mice and TNKS1BP1-/- mice after 1 month following exposing to IR. (F) quantitative of (E). (G) iNOS, α-SMA, P21 and P16 expression level on lung tissue of wild type mice and TNKS1BP1-/- mice after 2 month following exposing to IR. (H) quantitative of (G). Data are presented as mean ±SD. Statistical analysis was conducted by One-way ANOVA; *p<0.05, **p<0.01, ***p<0.001 mean the difference statistical significance.

Additional file 4: Supplementary Figure 3. TNKS1BP1 promoted epithelial cells senescence in RILI mice lung tissue. (A). The localization of SFTPC (shown in green) and P21 (shown in red) in lung tissues at 1 month after IR was determined by confocal microscopy. (B). The localization of SFTPC (shown in green) and P21 (shown in red) in lung tissues at 2 months after IR was determined by confocal microscopy. Cell nuclei were visualized by DAPI (shown in blue). White arrows indicate the co-location between SFPTC and P21. Scale bar, 100 μm.

Additional file 5: Supplementary Figure 4. TNKS1BP1 promoted macrophages activation in RILI mice lung tissue. (A). The localization of F4/80 (shown in green) and iNOS (shown in red) in lung tissues at 1 month after IR was determined by confocal microscopy. (B). The localization of F4/80 (shown in green) and iNOS (shown in red) in lung tissues at 2 months after IR was determined by confocal microscopy. Cell nuclei were visualized by DAPI (shown in blue). White arrows indicate the co-location between F4/80 and iNOS. Scale bar, 100 μm.

Additional file 6.

## Data Availability

No datasets were generated or analysed during the current study.
