# Supplementary material for: TNKS1BP1 mediates AECII senescence and radiation induced lung injury through suppressing EEF2 degradation
Source: Respir Res. 2024 Aug 7;25:299. doi: 10.1186/s12931-024-02914-y (PMC11308570; doi:10.1186/s12931-024-02914-y)
Supplement: Supplementary file 1 — Additional file 1. [file 12931_2024_2914_MOESM1_ESM.doc]

**Tables**

Supplementary Table 1 Primers used in this study

| Primer name | Sequence from 5'to3' |
| --- | --- |
| IL6 – F (human) | CACTGGTCTTTTGGAGTTTGAG |
| IL6 – R (human) | GGACTTTTGTACTCATCTGCAC |
| IL1β – F (human) | GCCAGTGAAATGATGGCTTATT |
| IL1β – R (human) | AGGAGCACTTCATCTGTTTAGG |
| β-actin F (human) | GGCTATGCTCTCCCTCACG |
| β-actin R (human) | CGCTCGGTCAGGATCTTCAT |

Supplementary Table 2 Antibodies used in the study

| Antibody | Cat. | Source |
| --- | --- | --- |
| TNKS1BP1 | sc-514517 | Santa Cruz |
| EEF2 | 2332S | Cell Signaling Technology |
| CNOT4 | sc-517324 | Santa Cruz |
| P21 | sc-6246 | Santa Cruz |
| P16 | ab51243 | Abcam |
| P53 | sc-126 | Santa Cruz |
| β-actin | TA-09 | ZSGB-BIO |
| GAPDH | TA-08 | ZSGB-BIO |
