# Supplementary figures and images for: TNKS1BP1 mediates AECII senescence and radiation induced lung injury through suppressing EEF2 degradation
Source: Respir Res. 2024 Aug 7;25:299. doi: 10.1186/s12931-024-02914-y (PMC11308570; doi:10.1186/s12931-024-02914-y)

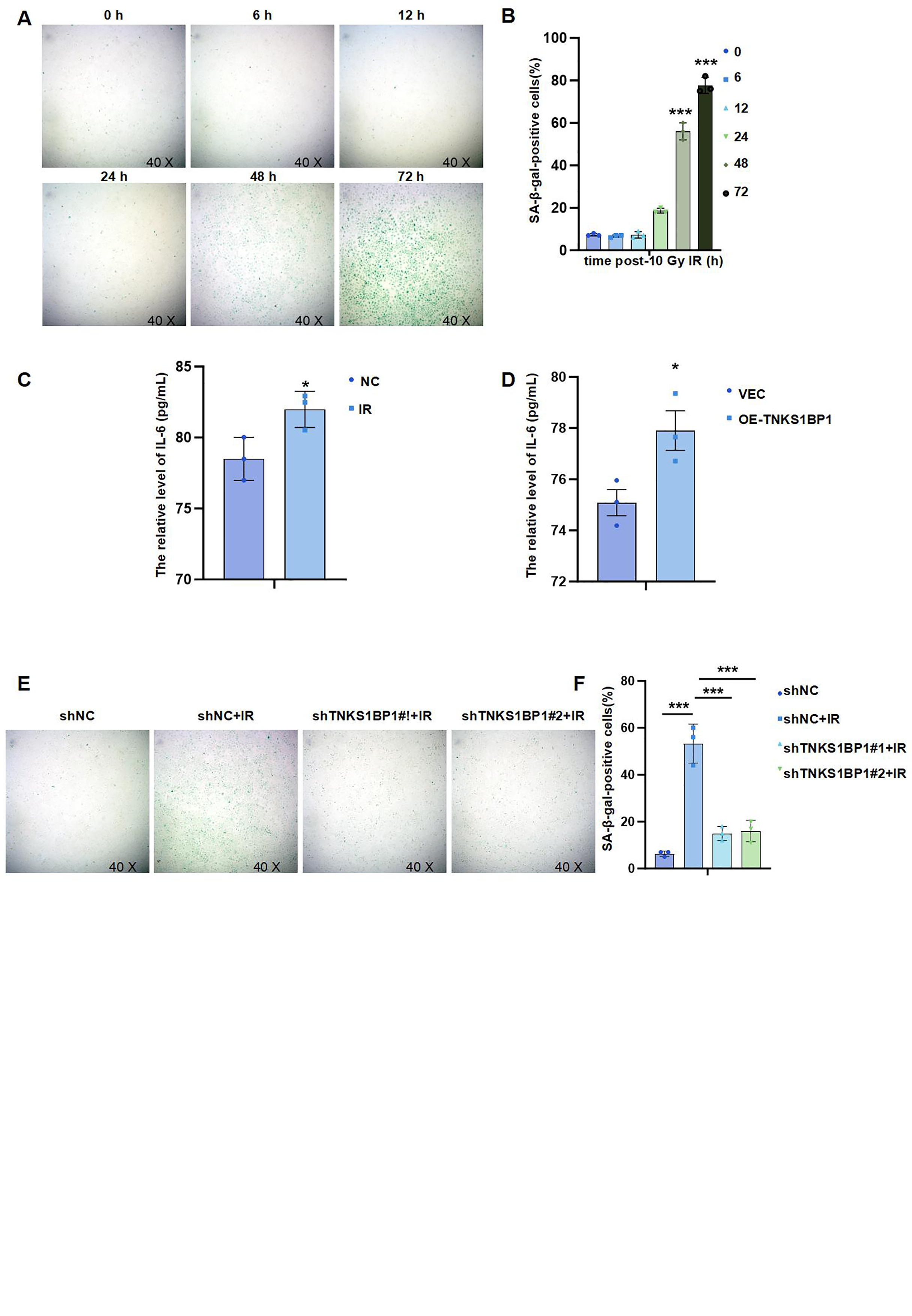

Supplement: Supplementary file 2 — Additional file 2: Supplementary Figure 1. The TNKS1BP1 promote IR induced AECII senescence. (A). SA-β-gal staining was performed on A549 cells at different time points (0 h, 6 h, 12 h, 24 h, 48 h, and 72 h) after exposure to 10 Gy of radiation; (B) The proportion of SA-β-gal-positive cells; (C). IL-6 levels detected by ELISA in the supernatants of A549 cells at 48 h after exposure to 10 Gy of radiation; (D) IL-6 levels detected by ELISA in the supernatants of A549 cells at 48 h after over-expression of TNKS1BP1. (E). SA-β-gal staining of A549 cells at each group; (F) The proportion of SA-β-gal-positive cells. Data are presented as mean ±SD. For statistical analysis, B, C and D were conducted by unpaired Student’s t test; F was conducted by One-way ANOVA; *p<0.05, ***p<0.001 mean the difference statistical significance. [file 12931_2024_2914_MOESM2_ESM.jpg]

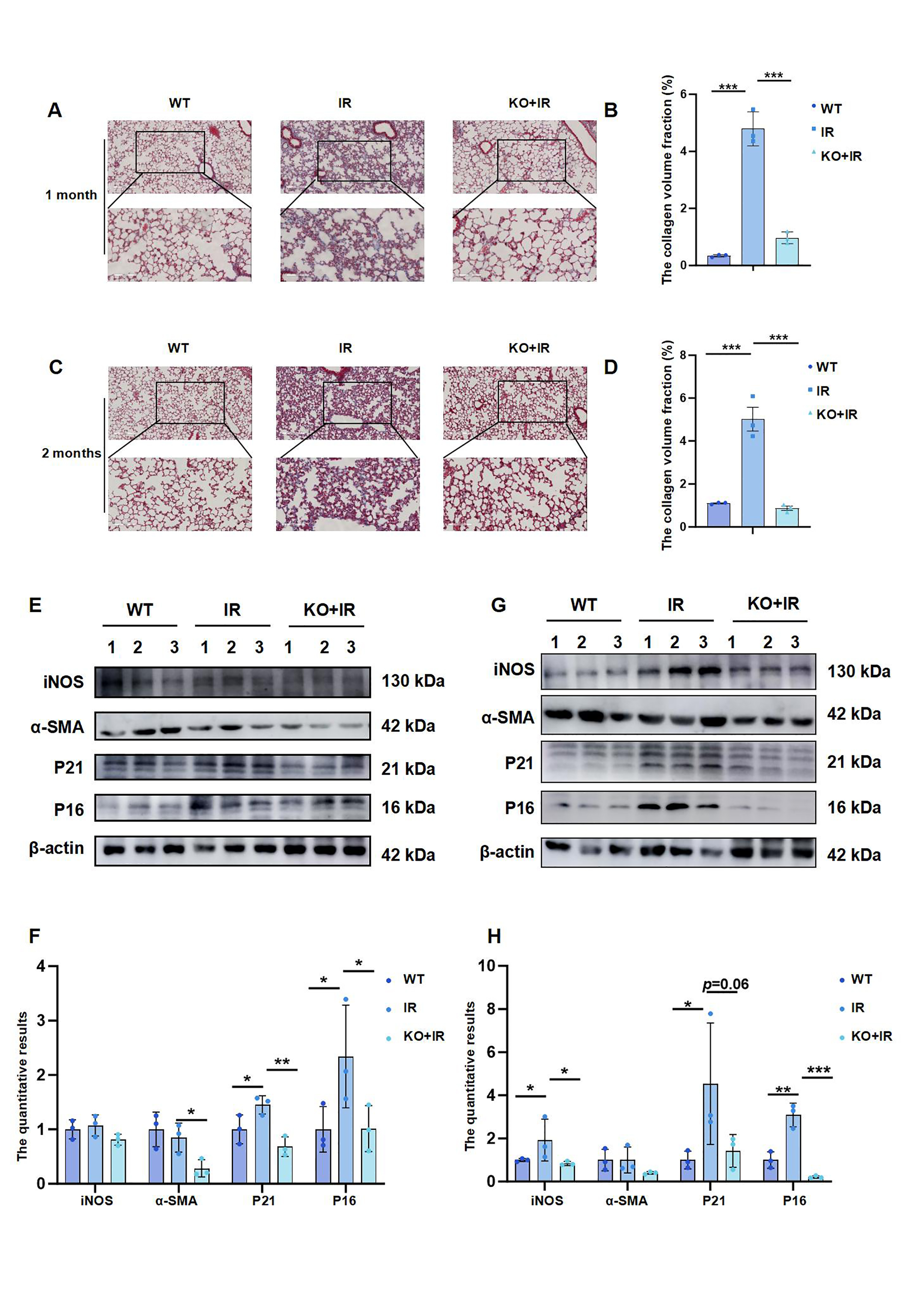

Supplement: Supplementary file 3 — Additional file 3: Supplementary Figure 2. TNKS1BP1 deletion ameliorates the IR induced lung tissue senescence. (A) Masson’s staining of lung tissues at 1 month after IR. Scale bar, 200 μm or 100 μm. (B) Collagen quantification by Masson’s trichrome staining of lung tissues with ImageJ software. (C) Masson’s staining of lung tissues at 2 months after IR. Scale bar, 200 μm or 100 μm. (D) Collagen quantification by Masson’s trichrome staining of lung tissues with ImageJ software. (E) iNOS, α-SMA, P21 and P16 expression level on lung tissue of wild type mice and TNKS1BP1-/- mice after 1 month following exposing to IR. (F) quantitative of (E). (G) iNOS, α-SMA, P21 and P16 expression level on lung tissue of wild type mice and TNKS1BP1-/- mice after 2 month following exposing to IR. (H) quantitative of (G). Data are presented as mean ±SD. Statistical analysis was conducted by One-way ANOVA; *p<0.05, **p<0.01, ***p<0.001 mean the difference statistical significance. [file 12931_2024_2914_MOESM3_ESM.jpg]

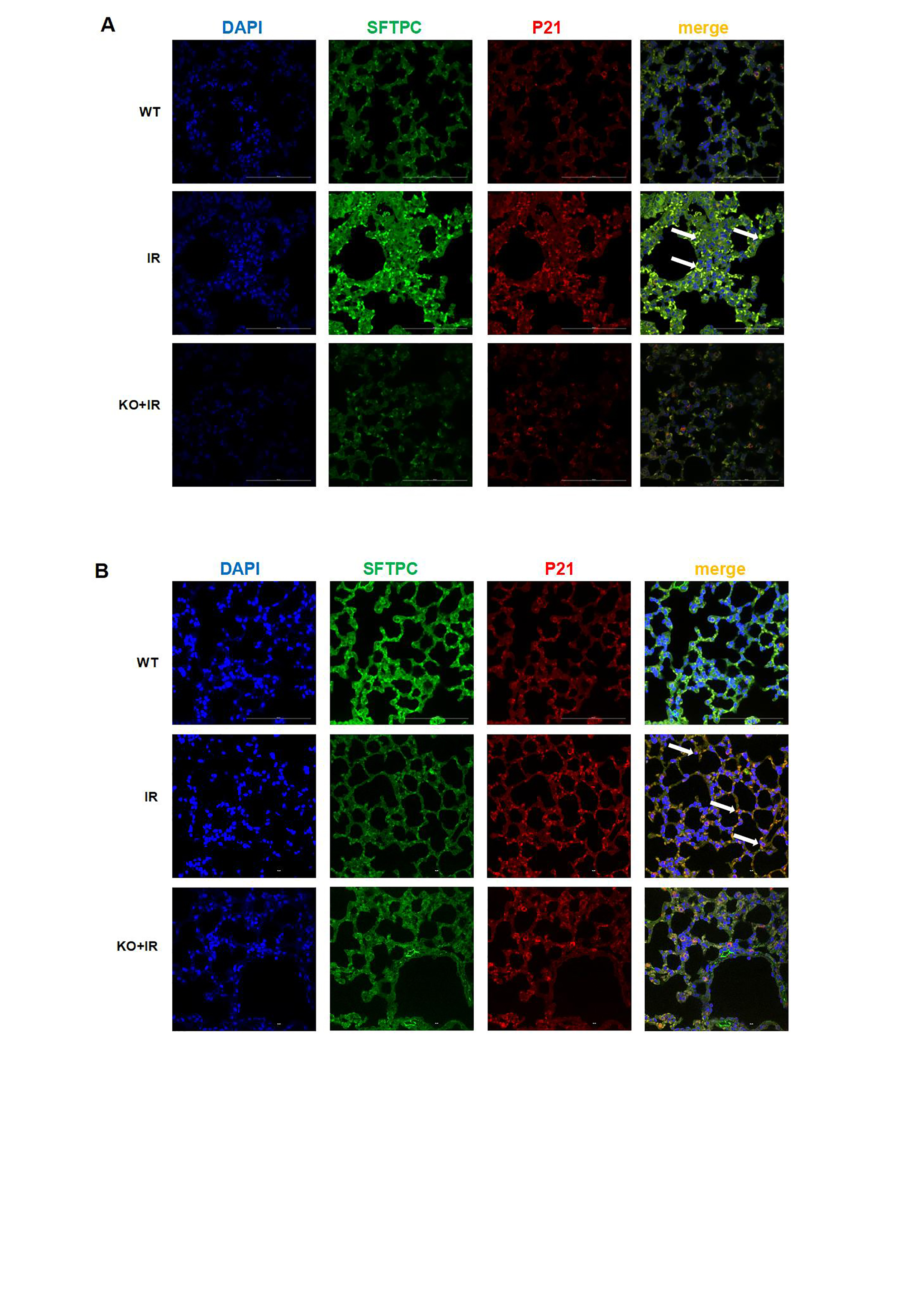

Supplement: Supplementary file 4 — Additional file 4: Supplementary Figure 3. TNKS1BP1 promoted epithelial cells senescence in RILI mice lung tissue. (A). The localization of SFTPC (shown in green) and P21 (shown in red) in lung tissues at 1 month after IR was determined by confocal microscopy. (B). The localization of SFTPC (shown in green) and P21 (shown in red) in lung tissues at 2 months after IR was determined by confocal microscopy. Cell nuclei were visualized by DAPI (shown in blue). White arrows indicate the co-location between SFPTC and P21. Scale bar, 100 μm. [file 12931_2024_2914_MOESM4_ESM.jpg]

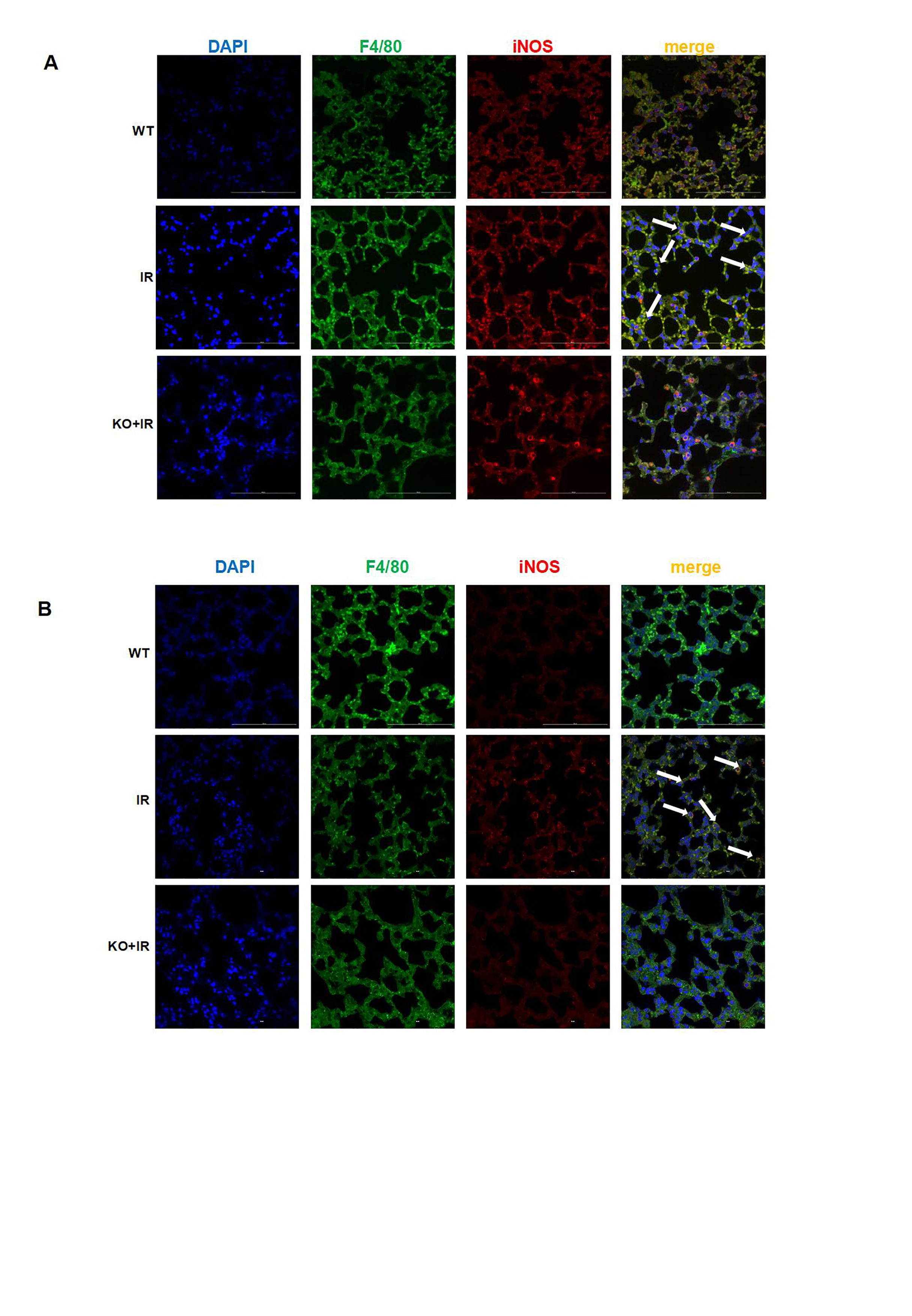

Supplement: Supplementary file 5 — Additional file 5: Supplementary Figure 4. TNKS1BP1 promoted macrophages activation in RILI mice lung tissue. (A). The localization of F4/80 (shown in green) and iNOS (shown in red) in lung tissues at 1 month after IR was determined by confocal microscopy. (B). The localization of F4/80 (shown in green) and iNOS (shown in red) in lung tissues at 2 months after IR was determined by confocal microscopy. Cell nuclei were visualized by DAPI (shown in blue). White arrows indicate the co-location between F4/80 and iNOS. Scale bar, 100 μm. [file 12931_2024_2914_MOESM5_ESM.jpg]
