# Supplementary material for: TNKS1BP1 mediates AECII senescence and radiation induced lung injury through suppressing EEF2 degradation
Source: Respir Res. 2024 Aug 7;25:299. doi: 10.1186/s12931-024-02914-y (PMC11308570; doi:10.1186/s12931-024-02914-y)
Supplement: Supplementary file 6 — Additional file 6. [file 12931_2024_2914_MOESM6_ESM.pdf]

figure 1

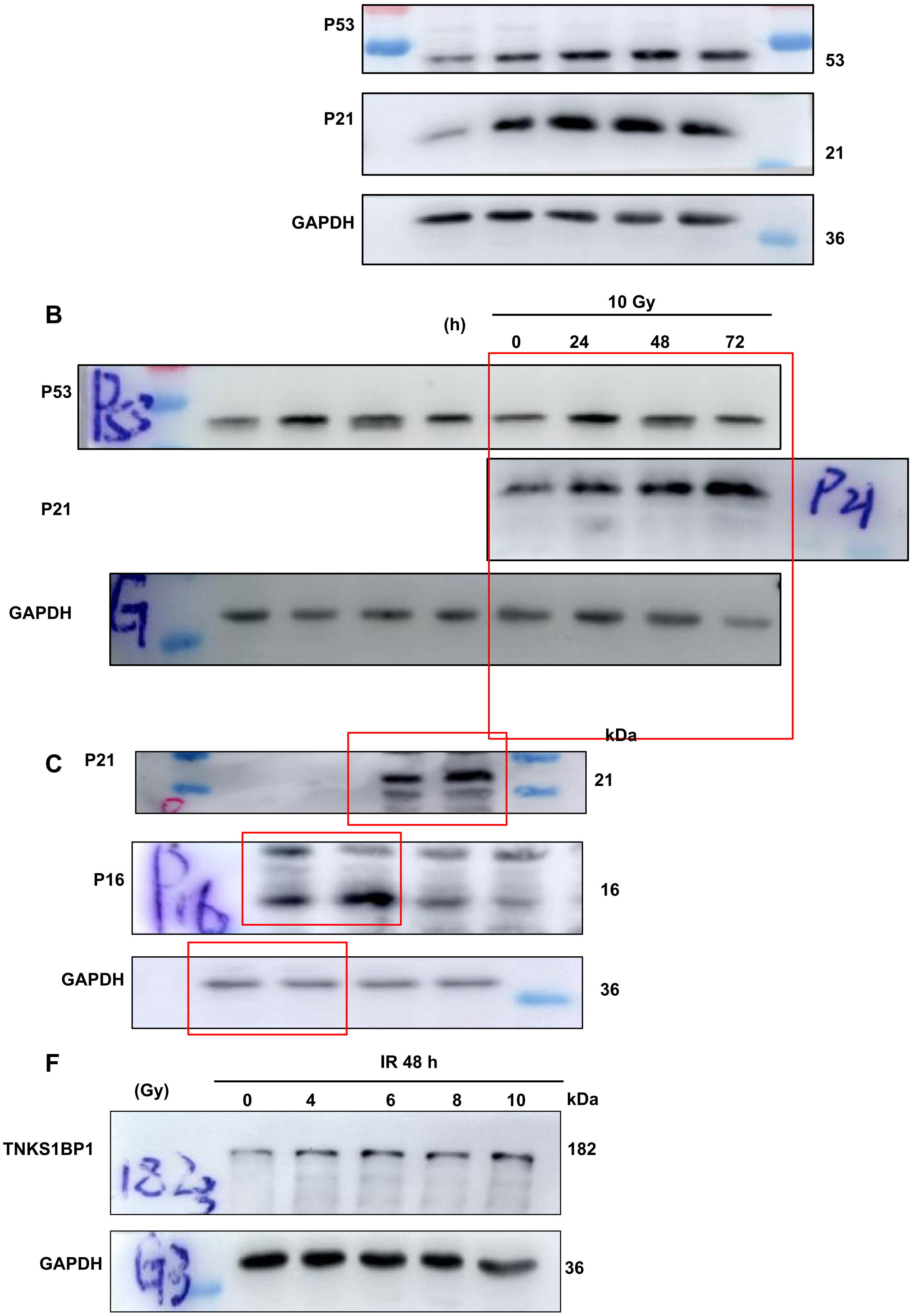
A
(Gy)
IR 48 h
0
4
6
8
10
kDa
P53
53
P21
21
GAPDH
36
B
10 Gy
(h)
0
24
48
72
P53
P21
GAPDH
C
P21
kDa
21
P16
16
GAPDH
36
F
IR 48 h
(Gy)
0
4
6
8
10
kDa
TNKS1BP1
182
GAPDH
36

figure 1

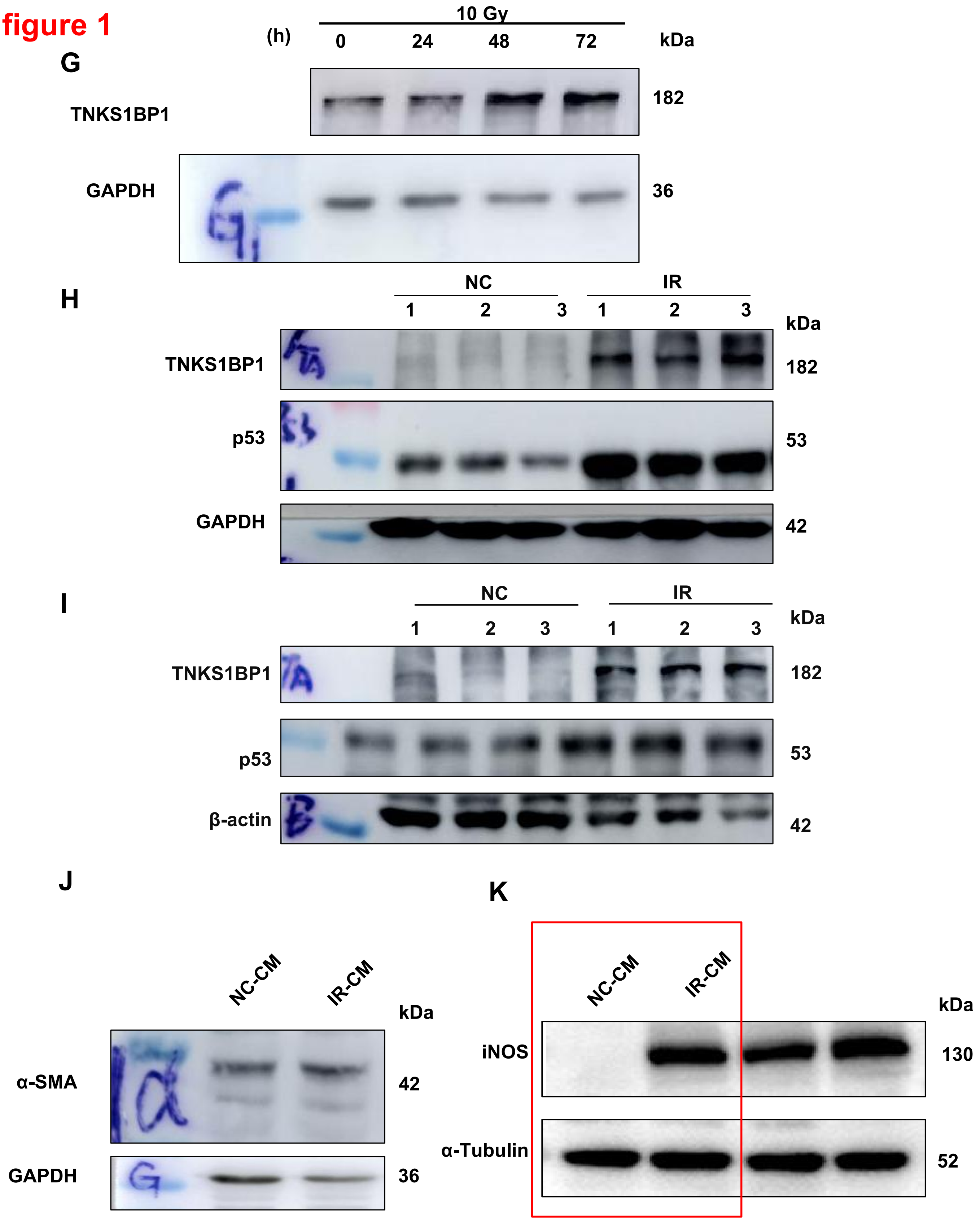

G
10 Gy
(h)
0
24
48
72
kDa
TNKS1BP1
182
GAPDH
36
H
NC
IR
1
2
3
1
2
3
kDa
TNKS1BP1
182
p53
53
GAPDH
42
I
NC
IR
1
2
3
1
2
3
kDa
TNKS1BP1
182
p53
53
β-actin
42
J
NC-CM
IR-CM
kDa
α-SMA
42
GAPDH
36
K
NC-CM
IR-CM
kDa
iNOS
130
α-Tubulin
52

figure 2

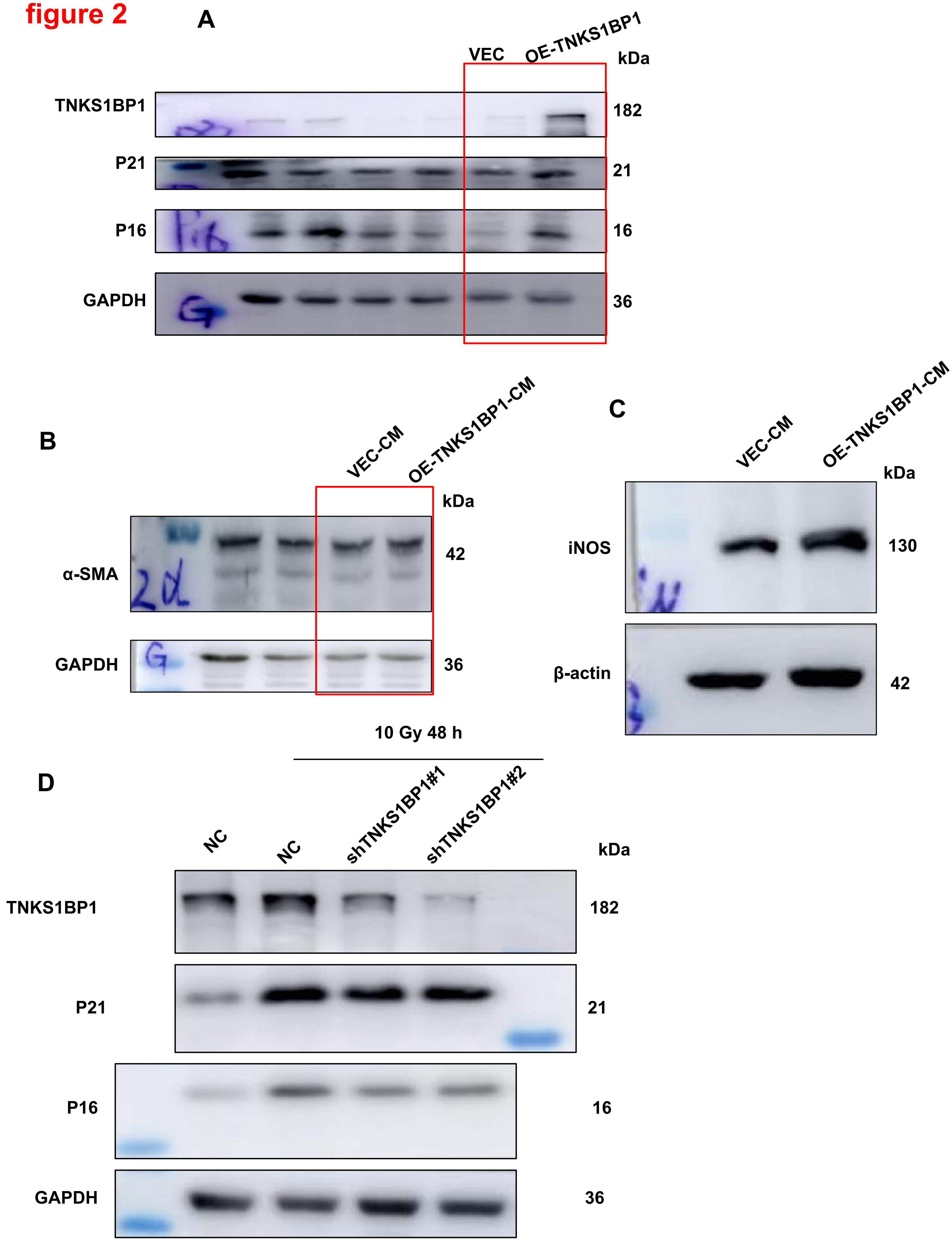
A
VEC
OE-TNKS1BP1
kDa
TNKS1BP1
182
P21
21
P16
16
GAPDH
36
B
VEC-CM
OE-TNKS1BP1-CM
kDa
α-SMA
42
GAPDH
36
C
VEC-CM
OE-TNKS1BP1-CM
kDa
iNOS
130
β-actin
42
10 Gy 48 h
D
NC
NC
shTNKS1BP1#1
shTNKS1BP1#2
kDa
TNKS1BP1
182
P21
21
P16
16
GAPDH
36

figure 2

G

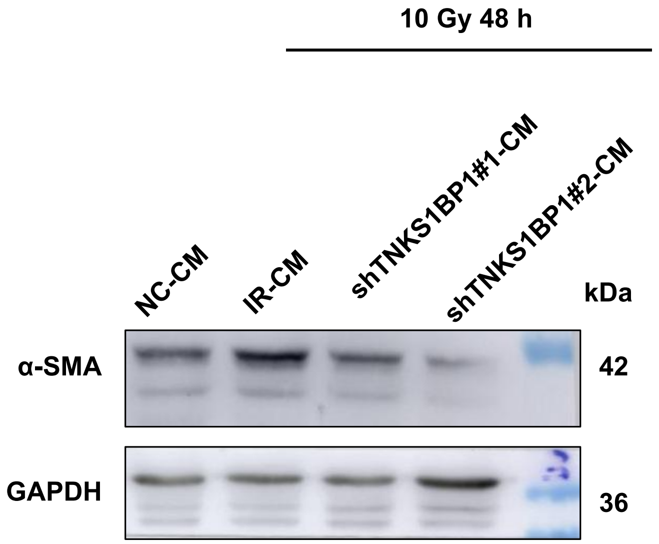
10 Gy 48 h
NC-CM
IR-CM
shTNKS1BP1#1-CM
shTNKS1BP1#2-CM
kDa
α-SMA
42
GAPDH
36

H

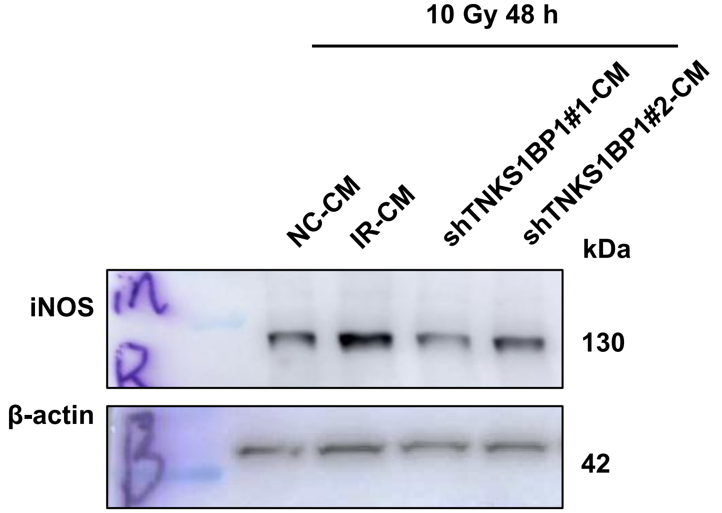
10 Gy 48 h
NC-CM
IR-CM
shTNKS1BP1#1-CM
shTNKS1BP1#2-CM
kDa
iNOS
130
β-actin
42

figure 3

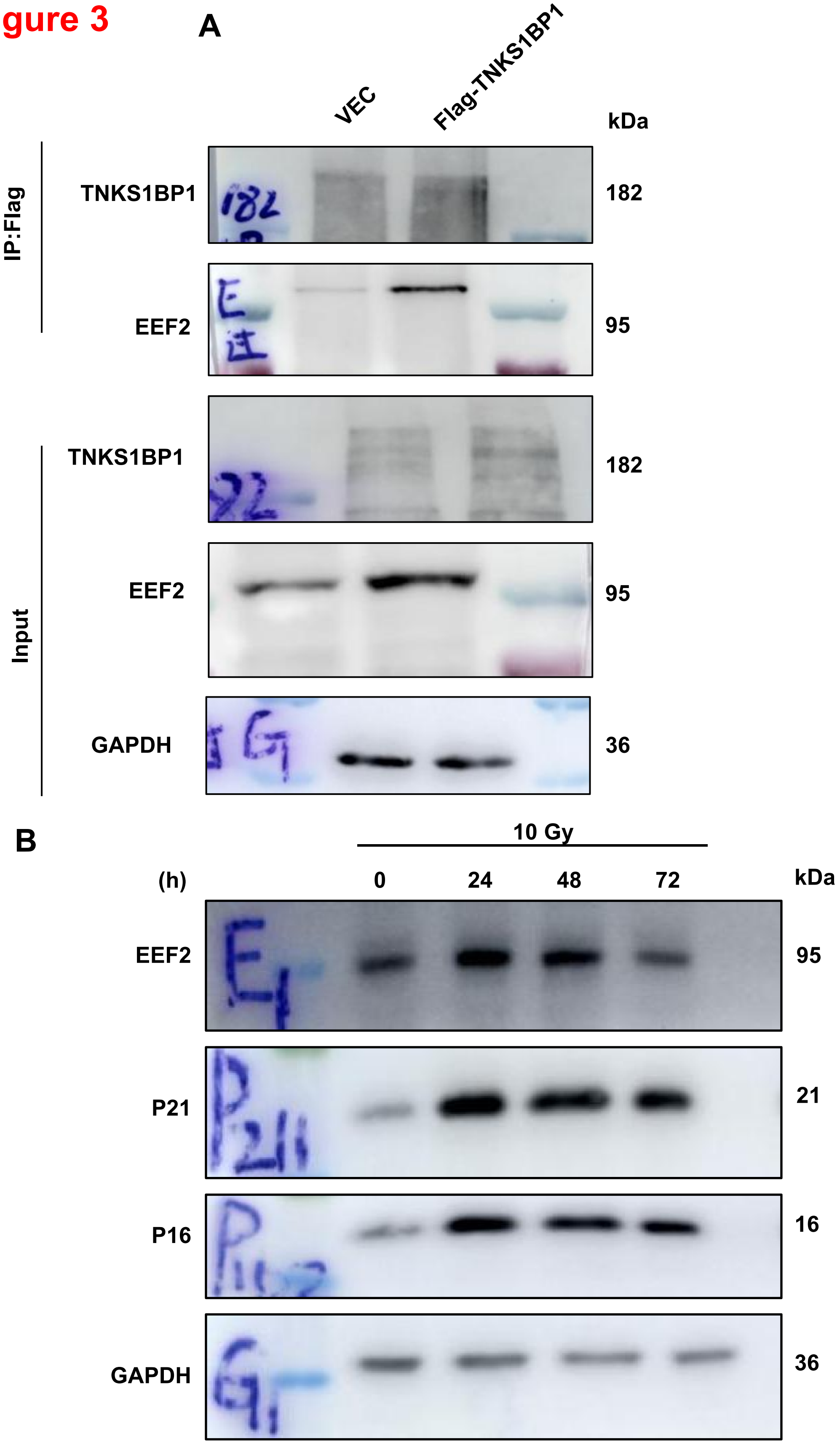
A
VEC
Flag-TNKS1BP1
kDa
IP:Flag
TNKS1BP1
182
EEF2
95
Input
TNKS1BP1
182
EEF2
95
GAPDH
36
B
10 Gy
(h)
0
24
48
72
kDa
EEF2
95
P21
21
P16
16
GAPDH
36

figure 3

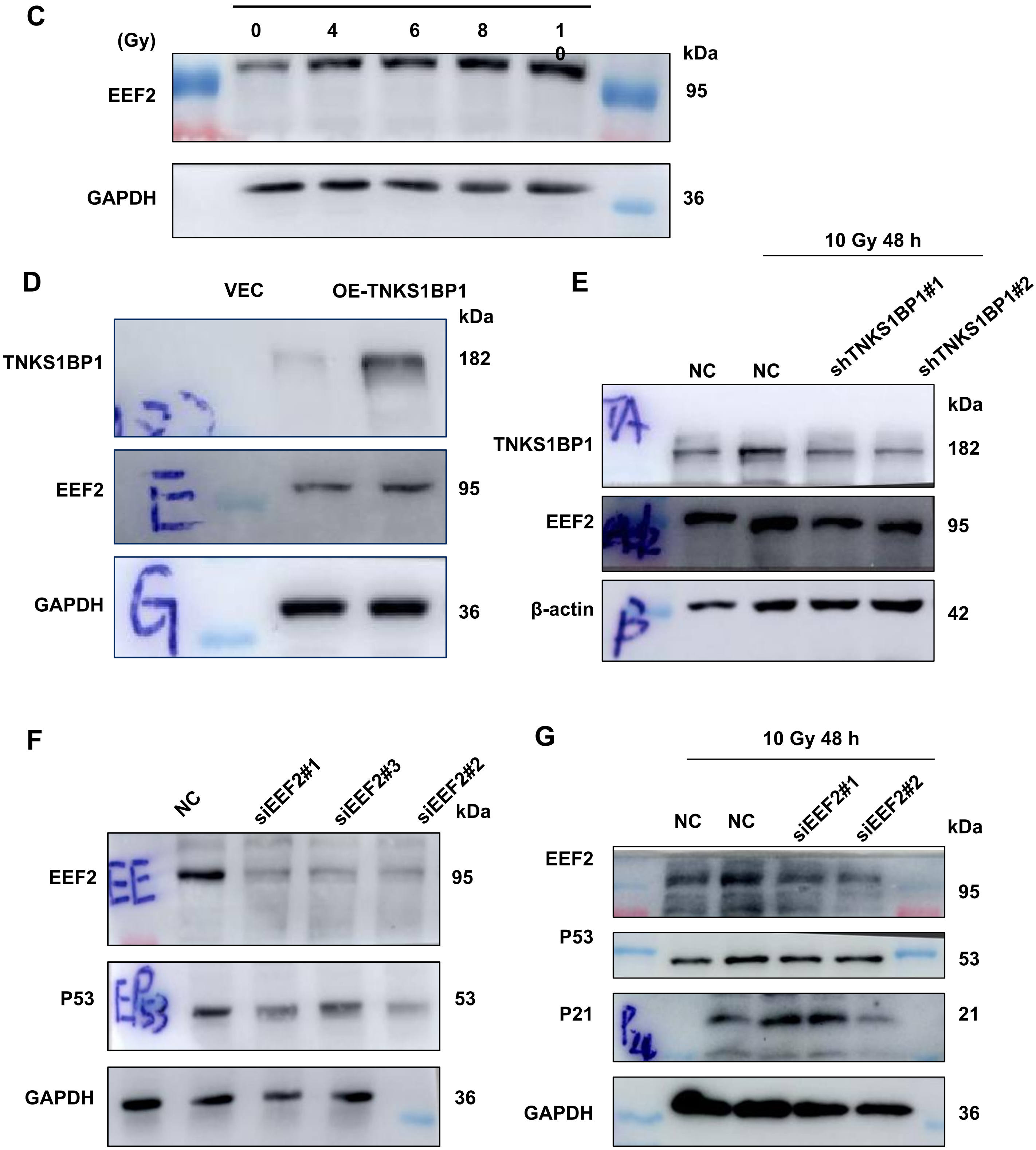

C
IR 48 h
(Gy)
0
4
6
8
10
kDa
EEF2
95
GAPDH
36
D
VEC
OE-TNKS1BP1
kDa
TNKS1BP1
182
EEF2
95
GAPDH
36
E
10 Gy 48 h
NC
NC
shTNKS1BP1#1
shTNKS1BP1#2
kDa
TNKS1BP1
182
EEF2
95
β-actin
42
F
NC
siEEF2#1
siEEF2#3
siEEF2#2
kDa
EEF2
95
P53
53
GAPDH
36
G
10 Gy 48 h
NC
NC
siEEF2#1
siEEF2#2
kDa
EEF2
95
P53
53
P21
21
GAPDH
36

figure 4

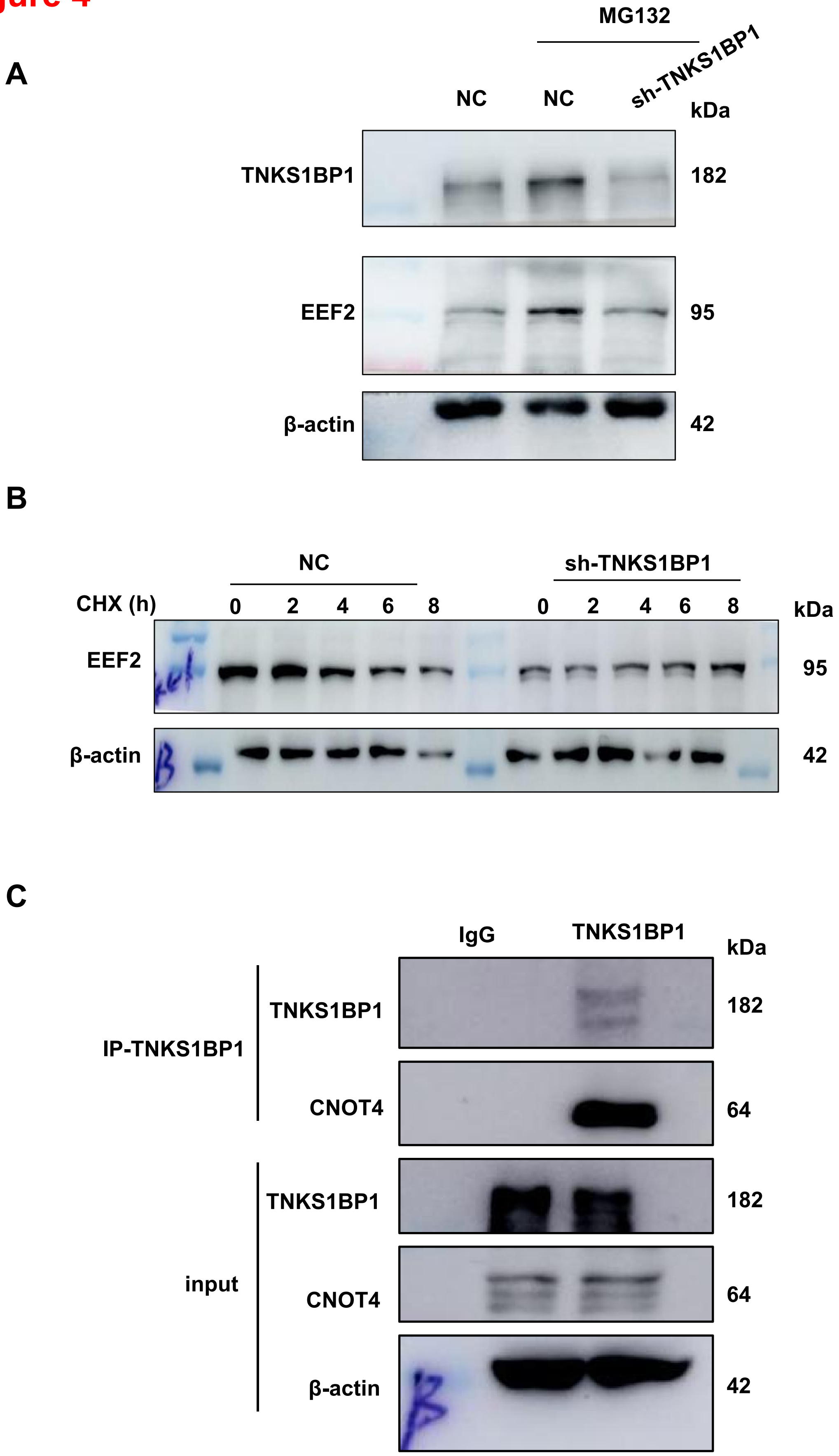
A
MG132
NC
NC
sh-TNKS1BP1
kDa
TNKS1BP1
182
EEF2
95
β-actin
42
B
NC
sh-TNKS1BP1
CHX (h)
0 2 4 6 8
0 2 4 6 8
kDa
EEF2
95
β-actin
42
C
IgG
TNKS1BP1
kDa
IP-TNKS1BP1
TNKS1BP1
182
CNOT4
64
input
TNKS1BP1
182
CNOT4
64
β-actin
42

figure 4

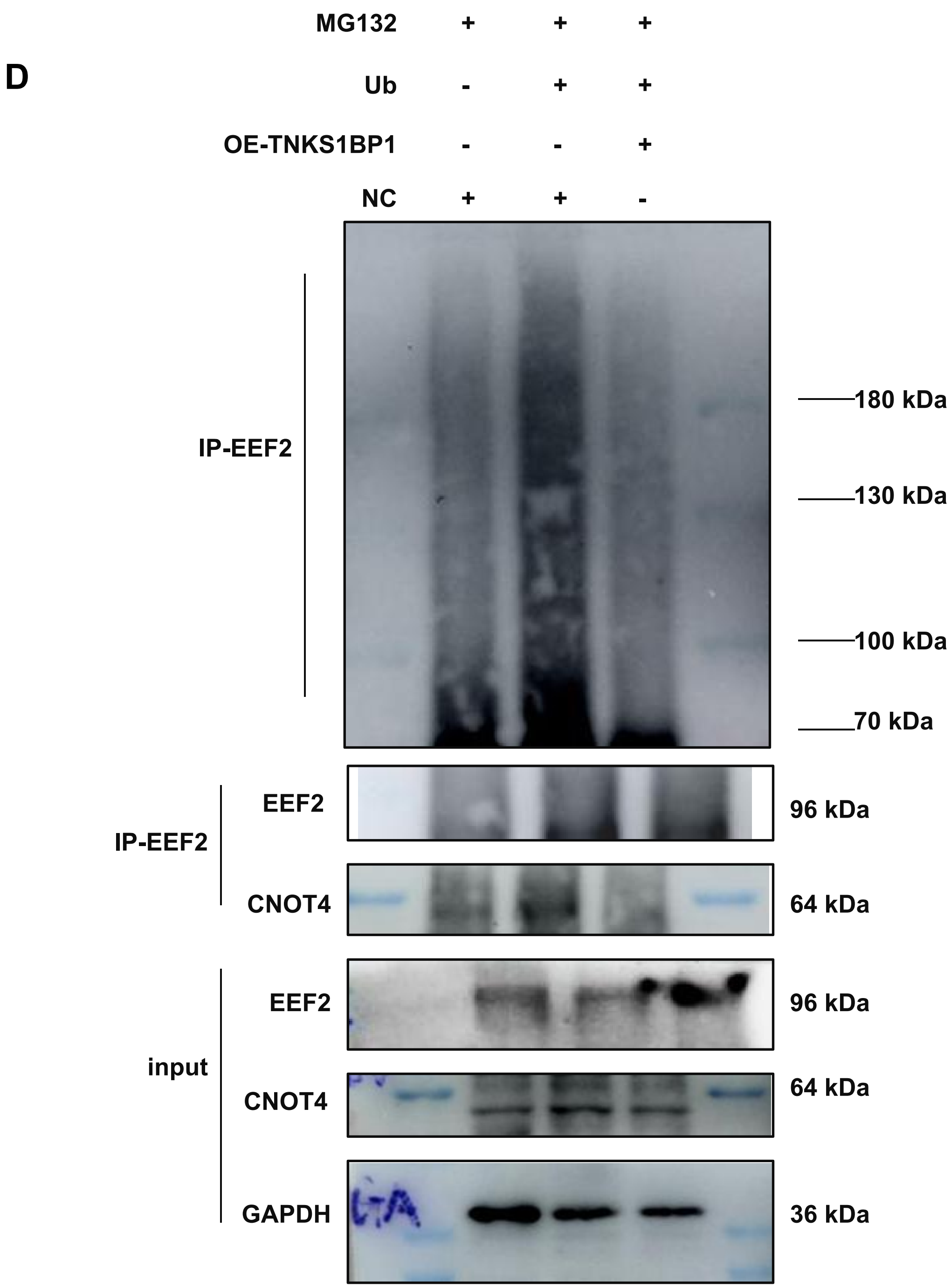

D
MG132 + + +
Ub - + +
OE-TNKS1BP1 - - +
NC + + -
IP-EEF2
180 kDa
130 kDa
100 kDa
70 kDa
IP-EEF2
EEF2
96 kDa
CNOT4
64 kDa
input
EEF2
96 kDa
CNOT4
64 kDa
GAPDH
36 kDa

figure 5

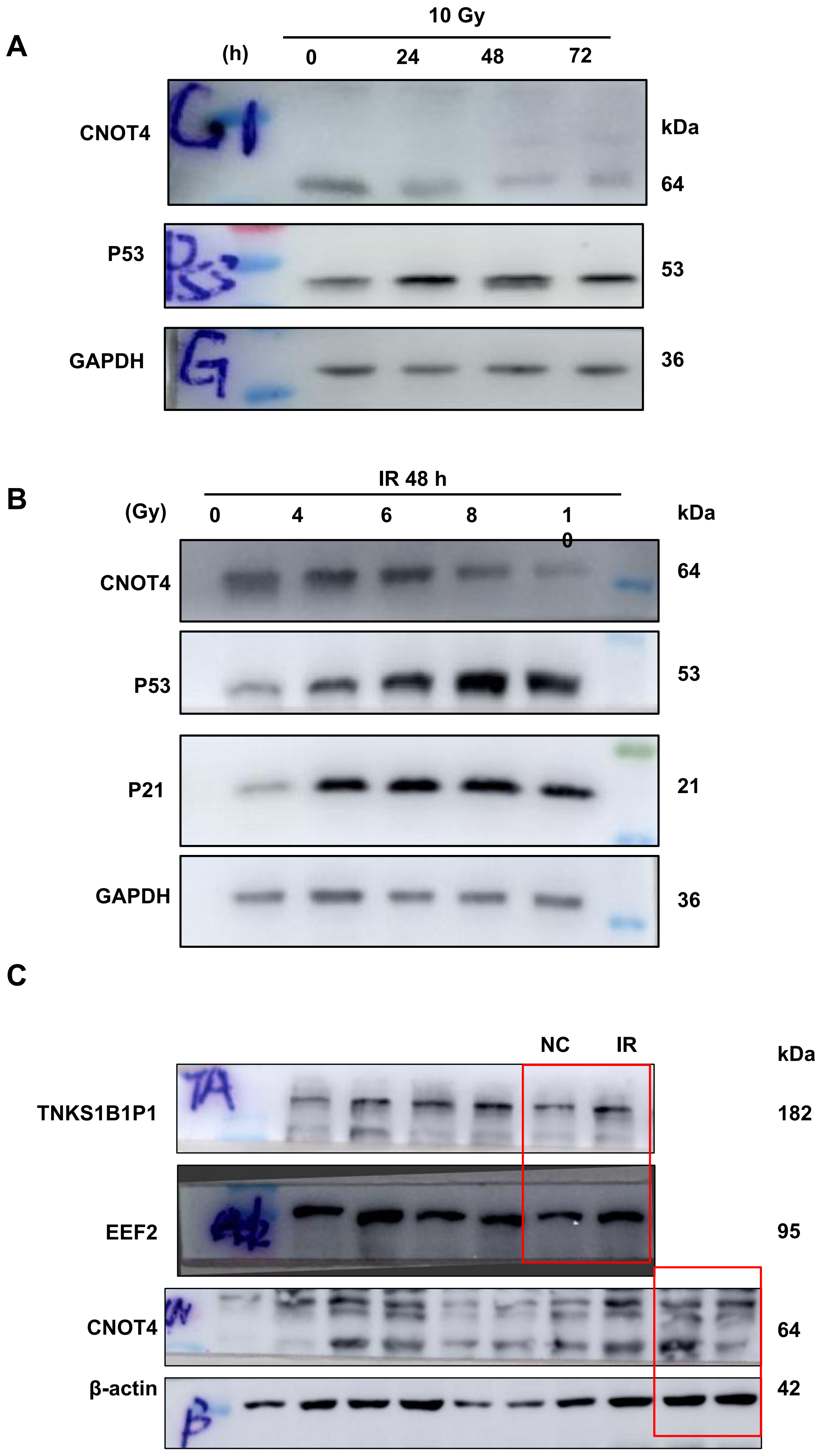
A
10 Gy
(h)
0
24
48
72
CNOT4
P53
GAPDH
kDa
64
53
36
B
IR 48 h
(Gy)
0
4
6
8
10
kDa
CNOT4
P53
P21
GAPDH
64
53
21
36
C
NC
IR
kDa
TNKS1B1P1
EEF2
CNOT4
β-actin
182
95
64
42

figure 5

D

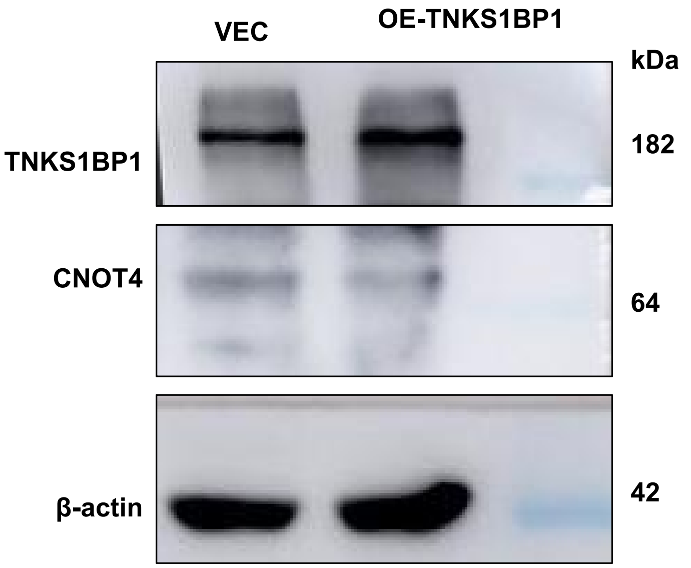
VEC
OE-TNKS1BP1
kDa
TNKS1BP1
182
CNOT4
64
β-actin
42

E

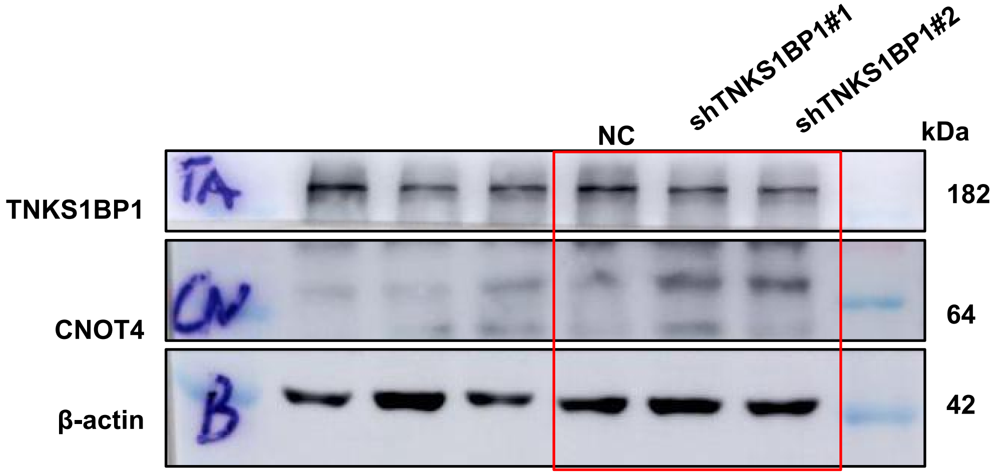
NC
shTNKS1BP1#1
shTNKS1BP1#2
kDa
TNKS1BP1
182
CNOT4
64
β-actin
42

F

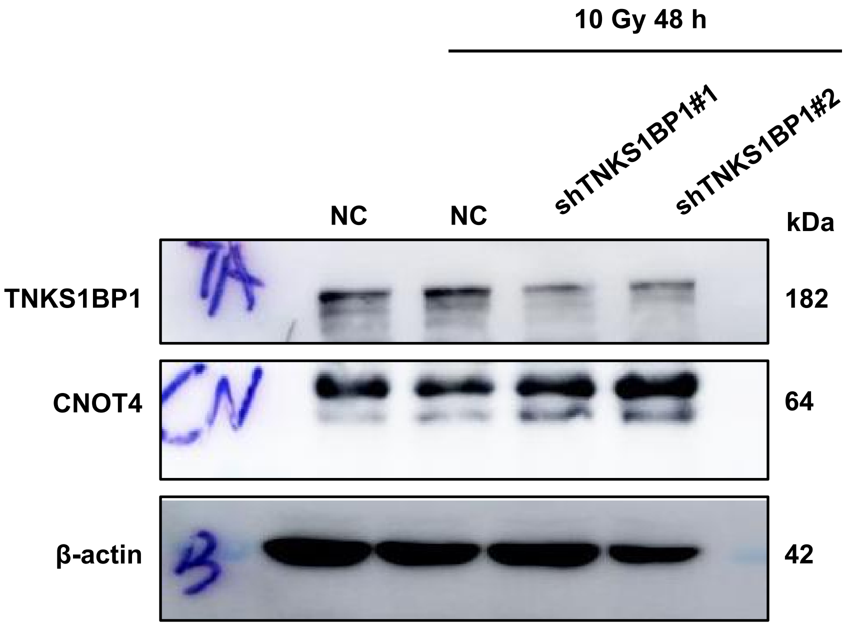
10 Gy 48 h
NC
NC
shTNKS1BP1#1
shTNKS1BP1#2
kDa
TNKS1BP1
182
CNOT4
64
β-actin
42

figure 5

G

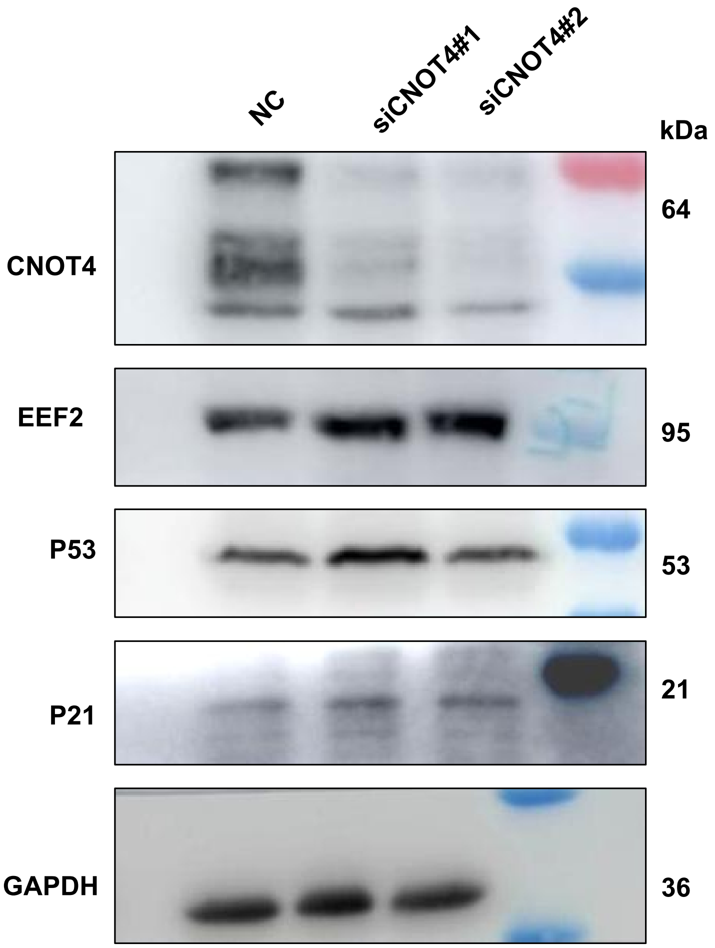
NC
siCNOT4#1
siCNOT4#2
kDa
CNOT4
64
EEF2
95
P53
53
P21
21
GAPDH
36

H

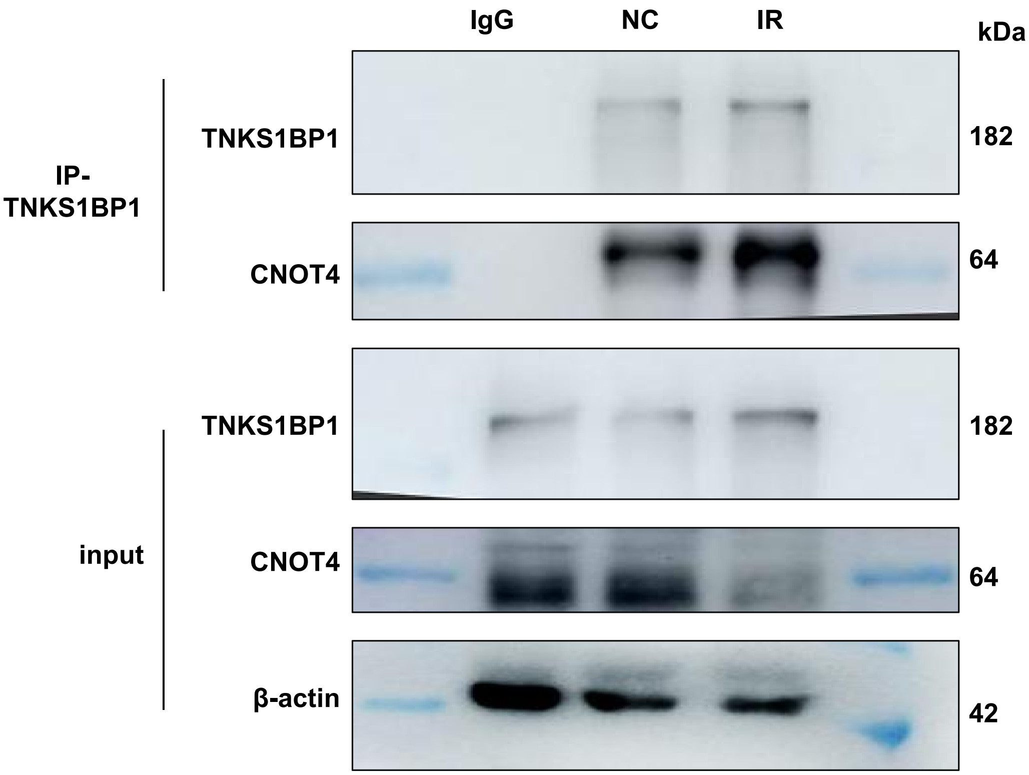
IgG
NC
IR
kDa
IP-
TNKS1BP1
TNKS1BP1
182
CNOT4
64
input
TNKS1BP1
182
CNOT4
64
β-actin
42

figure 6

A

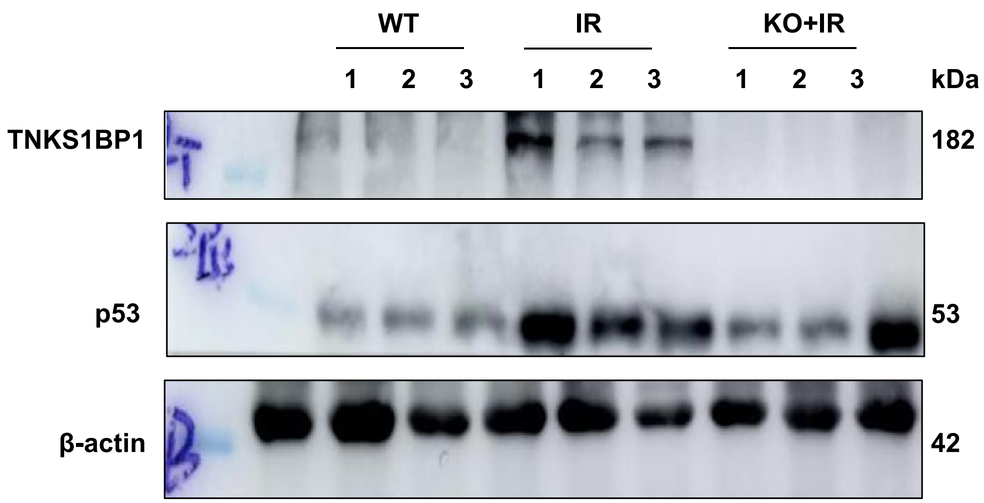
WT
IR
KO+IR
1 2 3 1 2 3 1 2 3
kDa
TNKS1BP1
182
p53
53
β-actin
42

B

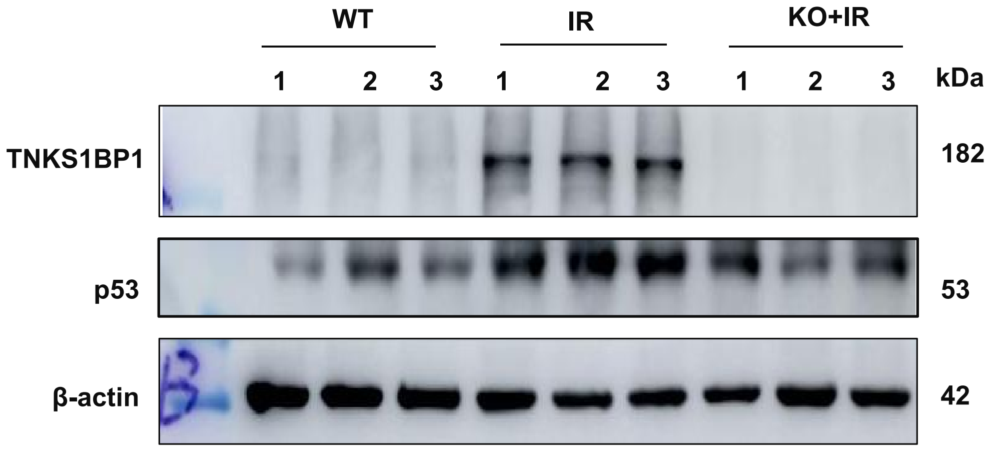
WT
IR
KO+IR
1 2 3 1 2 3 1 2 3
kDa
TNKS1BP1
182
p53
53
β-actin
42
